# Supplementary material for: Copy number signatures predict chromothripsis and clinical outcomes in newly diagnosed multiple myeloma
Source: Nat Commun. 2021 Aug 27;12:5172. doi: 10.1038/s41467-021-25469-8 (PMC8397708; doi:10.1038/s41467-021-25469-8)
Supplement: Supplementary file 1 — Supplementary Information [file 41467_2021_25469_MOESM1_ESM.pdf]

Copy number signatures predict chromothripsis and clinical outcomes in newly diagnosed multiple myeloma

Supplementary Figures and Tables

Maclachlan et al.

Supplementary Figures

Supplementary Figure 1. Chromothripsis tends to be stable in multiple myeloma patients at relapse. a) A longitudinal comparison of paired samples demonstrates that the majority of chromothripsis (purple) are conserved between diagnosis and post-treatment relapse. **b**) The cosine similarity of multiple myeloma copy number features across time is high (>0.9 in the majority), with exceptions seen in three patients who acquired a whole genome duplication at relapse (*) and in 1 sample having lost 3 large complex events (#). Both the number (**c**) and percentage (**d**) of conserved chromothripsis events is high compared with other structural variants (a; blue = chromoplexy, brown = templated insertions, orange = complex events not otherwise specified, c/d; green = acquired, orange = lost, blue = shared).

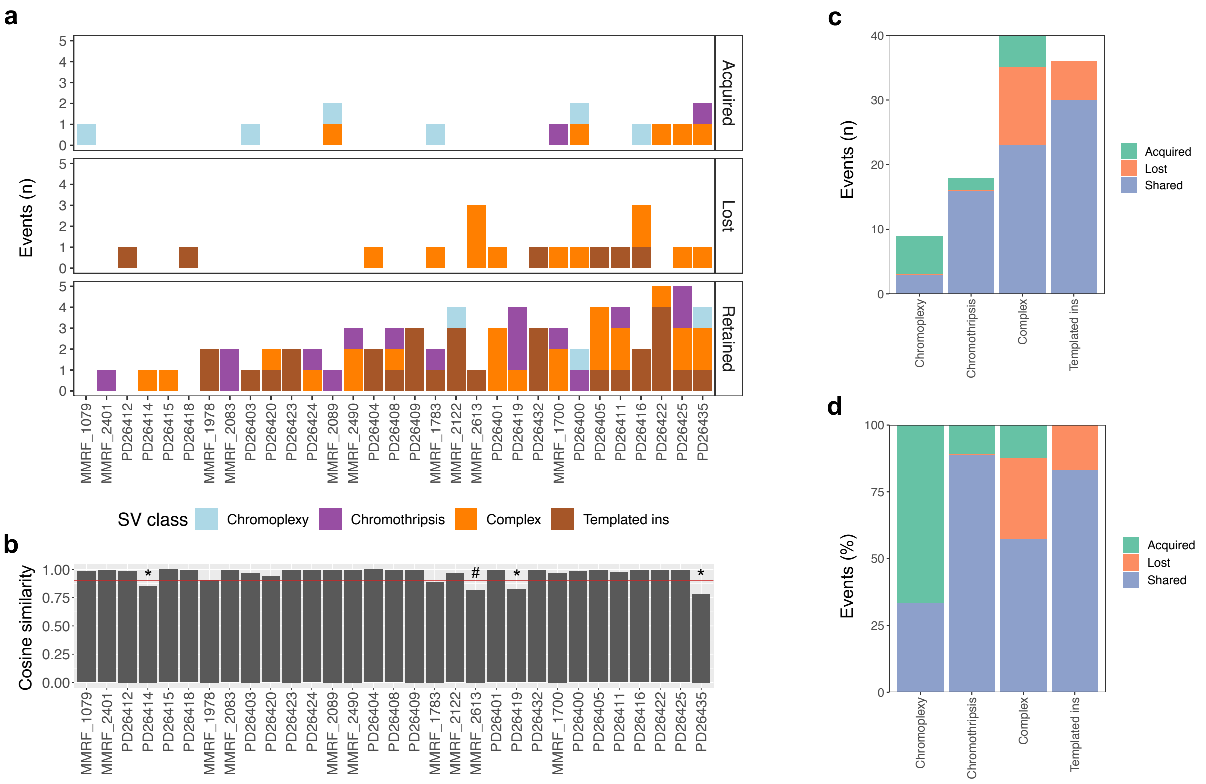

Supplementary Figure 2. The complexity of chromothripsis is lower in multiple myeloma (MM) and lymphoid cancers than in solid organ cancers. a) The maximum number of copy number gains within chromothripsis is lower in multiple myeloma and lymphoid cancers (red) than in the majority of solid organ cancers from the Pan-Cancer Analysis of Whole Genomes dataset (blue, restricted to histologies having >30 cases). **b**) The number of breakpoints due to chromothripsis per patient is lower in MM and lymphoid cancers than in the majority of solid organ cancers. In both (**a**) and (**b**) tumor types are ordered according to the median. Boxplots show the median and interquartile range (IQR), with whiskers extending to 1.5 * IQR. N = 23 for lymphoid cancers, 209 for MM, 925 for PCAWG data.

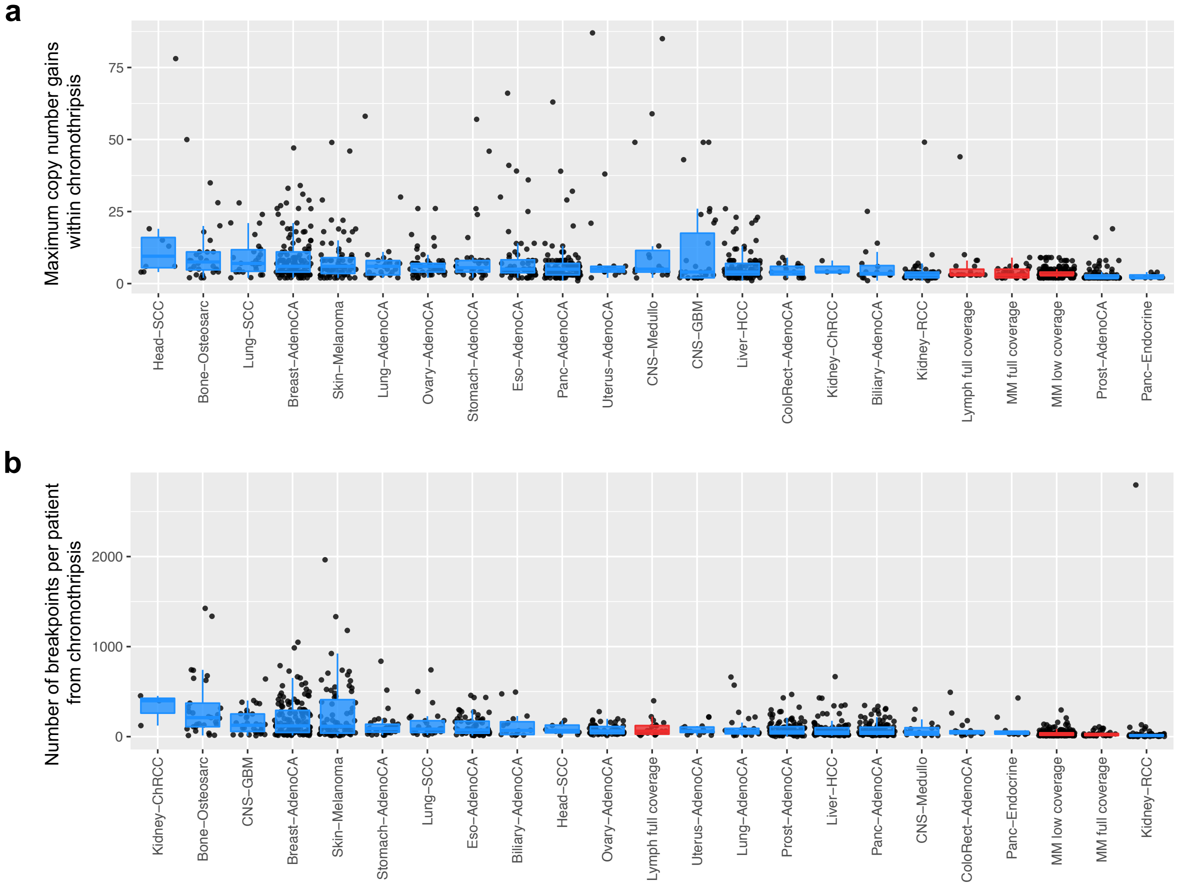

Supplementary Figure 3. Correlation of copy number (CN) signatures with t(11;14) and complex structural variant events. a) The contribution of CN-SIG4-5 is higher in those samples lacking t(11;14)(*CCDN1*;*IGH*) than in samples containing this translocation ($p = 3.2e^{-11}$). **b**) The contribution of CN-SIG4-5 is higher in those samples containing complex SV change, not otherwise specified (NOS) than in those without ($p = 2.2e^{-16}$). Boxplots show median and interquartile range IQR), with whiskers extending to 1.5 * IQR, p-values indicate significance by a 2-sided Wilcoxon rank sum test. (CN-SIG: copy number signature, pos=containing the feature, neg=without the feature).

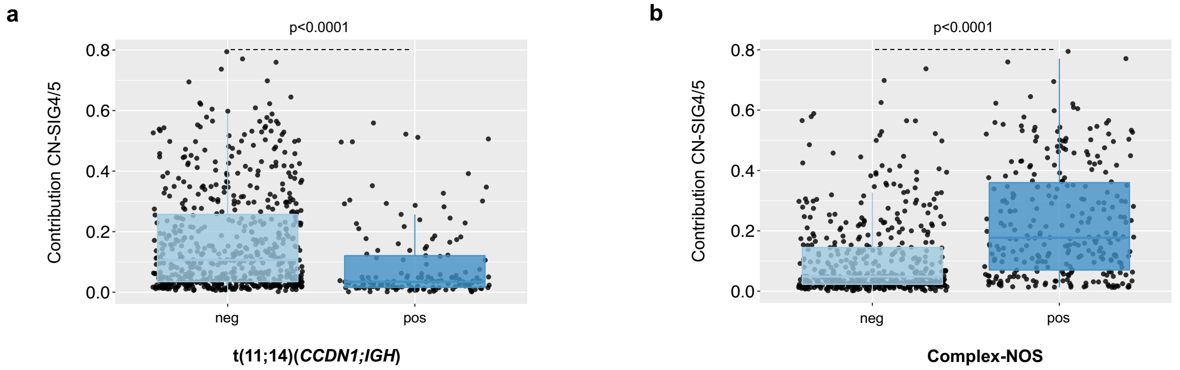

Supplementary Figure 4. Clinical data demonstrates the correlation of copy number (CN) and structural variant (SV) signatures with high-risk multiple myeloma prognostic features and complex genomic change. a) A heatmap of MM mutational and structural features demonstrates that contribution from CN-SIG4-5 and SV-SIG1-3 cluster with features of high-risk MM and with chromothripsis. Presence of biallelic *TP53* inactivation and chromosome 1q21 amplification (i.e. >3 copies) are annotated in dark red; presence of chromothripsis in purple; all the other genomic features are in bright red when present. (CN-SIG: copy number signature).

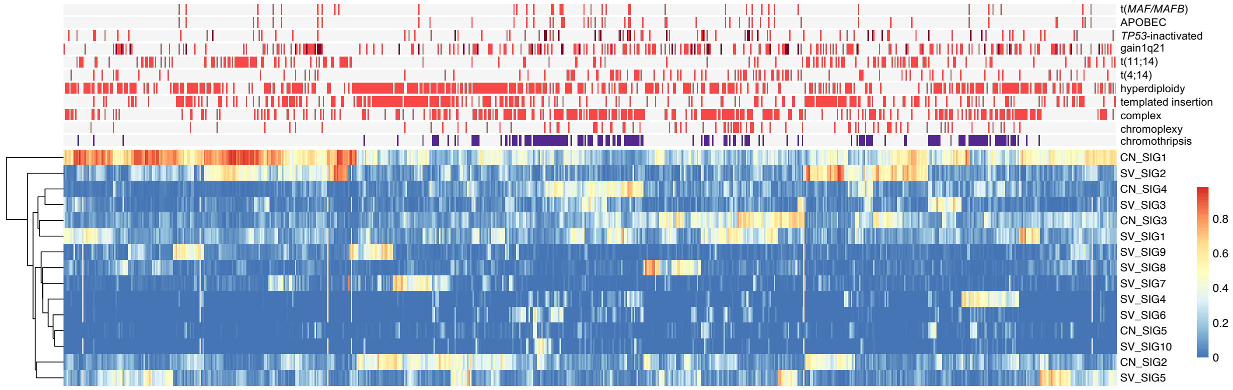

Supplementary Figure 5. Copy number (CN) signatures are less predictive of chromoplexy and complex structural variants (SV) compared with chromothripsis. Receiver operating curve (ROC) for the prediction of (**a**) chromoplexy and (**b**) complex SV (not otherwise specified) from CN signature analysis on CoMMpass whole genome sequencing (WGS) data (n=752). (Blue lines: individual ROC from 10-fold cross validation, red lines: mean of individual ROC, AUC: mean area-under-the-curve).

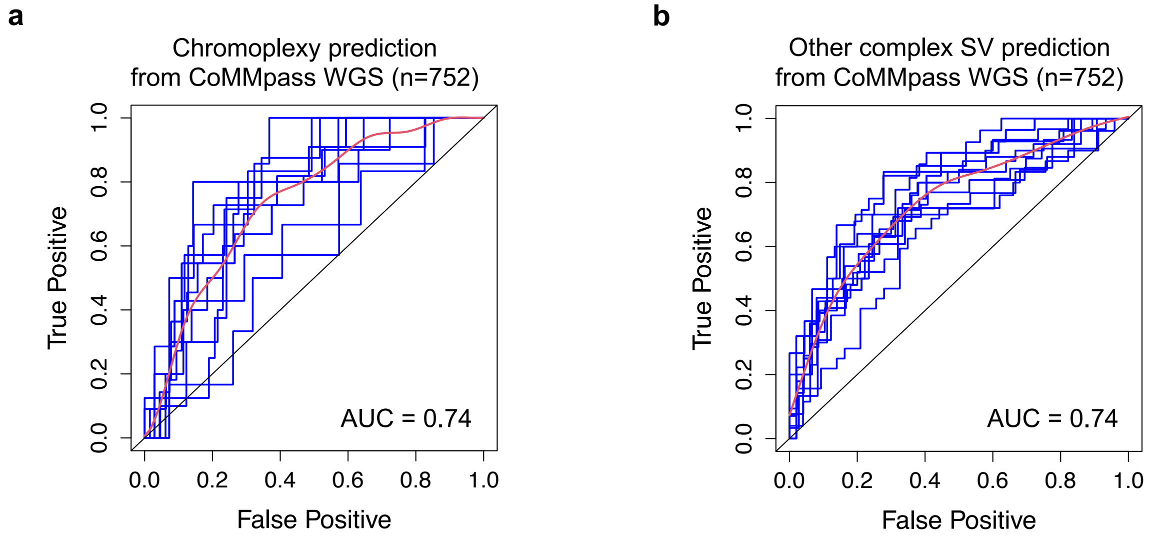

Supplementary Figure 6. Chromothripsis complexity contributes to detection by copy number (CN) signature analysis. a) A heatmap of complex structural features and the 28 CN features along with discrepancy in chromothripsis prediction by CN signatures. Neg/neg = no chromothripsis by manual curation and chromothripsis not predicted by CN signatures (grey), neg/pos = no chromothripsis but predicted by CN signatures (blue), pos/neg = chromothripsis present but not predicted by CN signatures (red) and pos/pos = chromothripsis present and predicted by CN signatures (purple). mb_10 = breakpoints per 10mb, count_cnv = absolute CN state, jump = change between adjacent breakpoints, band = breakpoints per chromosomal arm, osci = oscillation, size_cnv = segment size. **b**) A boxplot of the number (#) of chromosomal breakpoints attributed to chromothripsis by discrepancy in prediction (p = $9.6e^{-14}$, $1.9e^{-13}$, $7.1e^{-8}$). **c**) A boxplot of the maximum CN outside of chromothripsis (CT) by discrepancy in prediction. **d**) A boxplot of the number of chromosomal breakpoints outside of chromothripsis by discrepancy in prediction (p = 0.004, $5e^{-8}$, $6.1e^{-6}$, 0.019). All p-values indicate significance by a 2-sided Wilcoxon rank sum test, with all p-values being non-significant in (**c**), and (**d**) demonstrating comparisons with false positive and negative categories, n = 752.

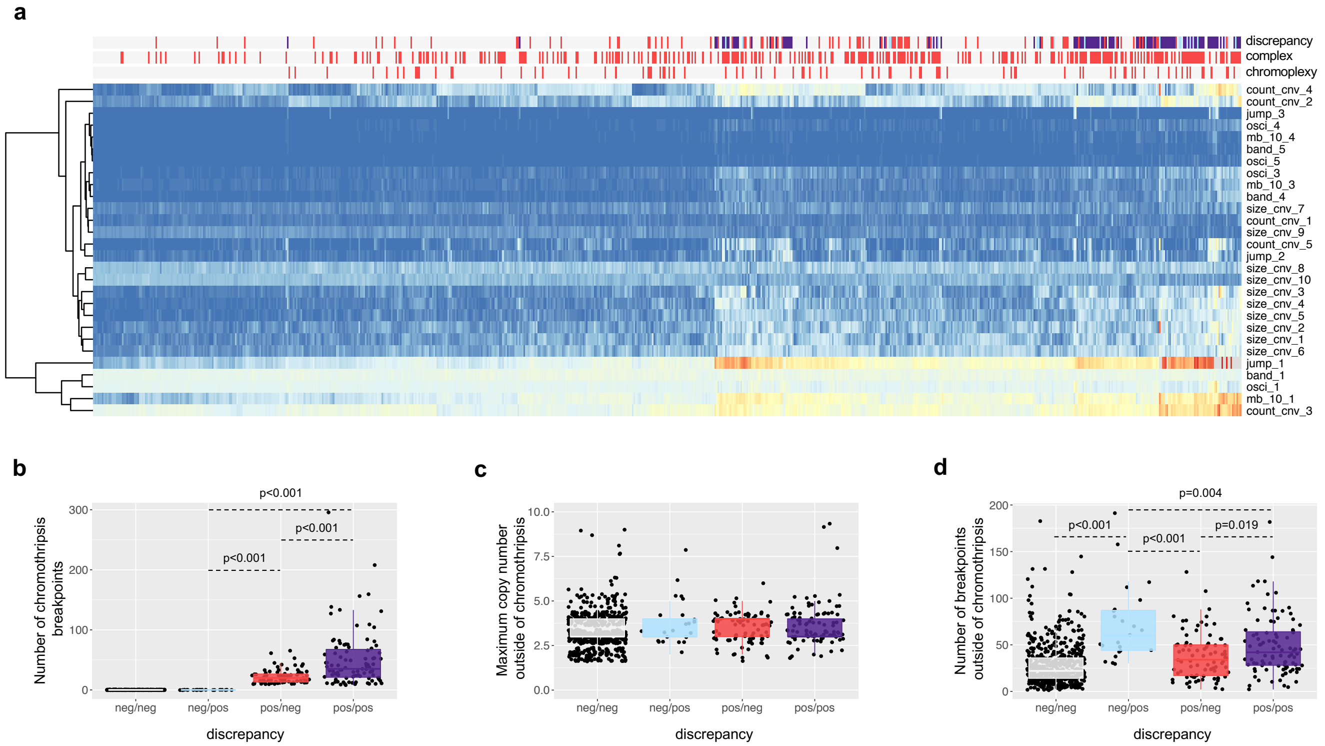

Supplementary Figure 7. The copy number (CN) and structural variant (SV) profile outside of chromothripsis lacks features of complexity. (**a-c**) The 28 CN feature profile from (**a**) genomic regions within chromothripsis, (**b**) samples containing chromothripsis, excluding chromothripsis areas, and (c) samples lacking chromothripsis. The cosine similarity between (a) and (b) is 0.75, while between (b) and (c) is 0.98. (**d-f**) The 32 SV feature profile from (**d**) genomic regions within chromothripsis, (**e**) in samples containing chromothripsis, excluding chromothripsis areas, and (**f**) samples lacking chromothripsis. The cosine similarity between (d) and (e) is 0.59, while between (b) and (c) is 0.96.

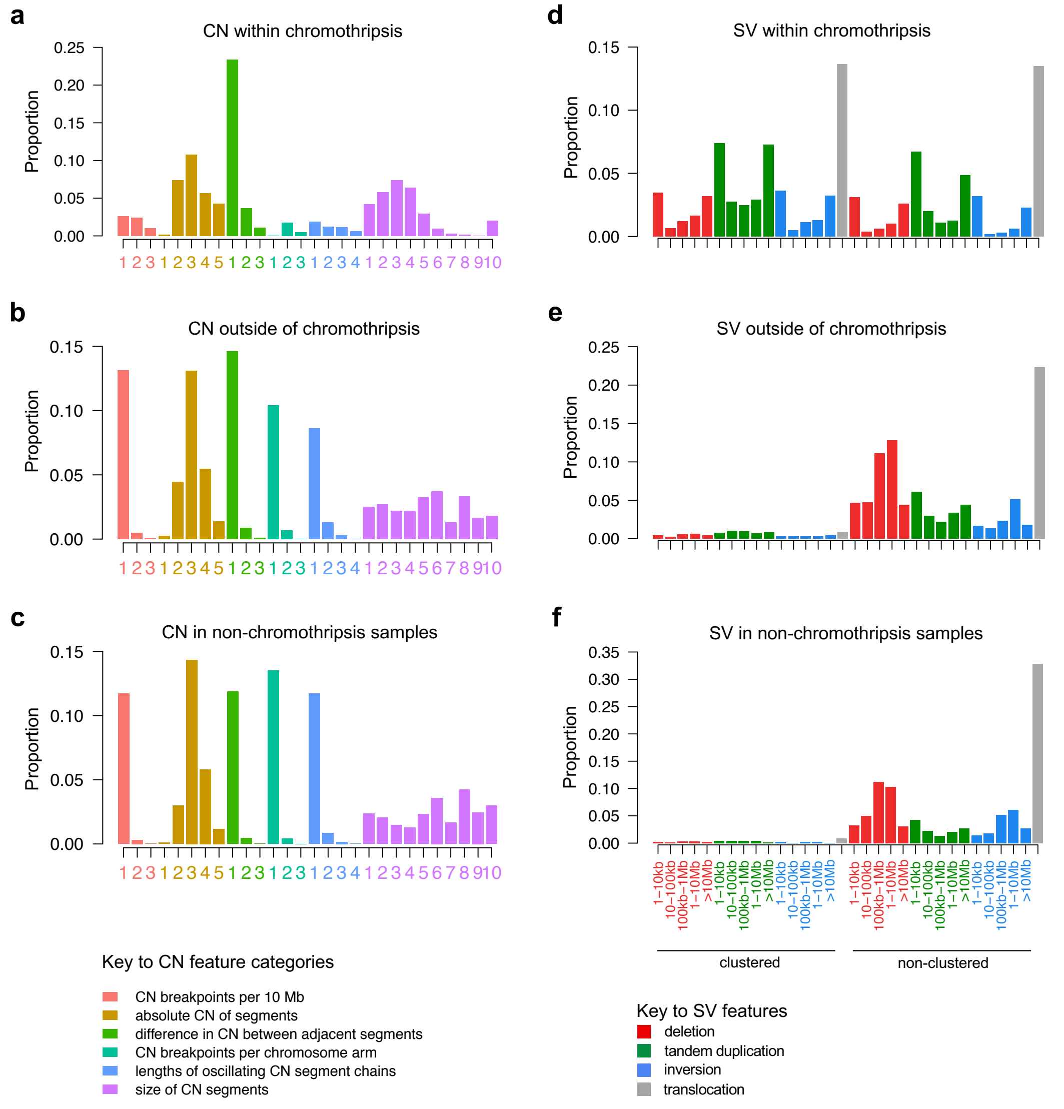

Supplementary Figure 8. Copy number signature landscape from a validation set of whole genomes from hematological cancers. a) Using *mclust* to define the optimum number of categories in each of the 6 copy number classes defined 26 categories in the validation set of whole genome sequencing (WGS), which contains multiple myeloma samples along with chronic lymphocytic leukemia, acute myeloid leukemia, and B-cell lymphoma. **b**) *De novo* extraction from the validation dataset extracted 4 CN signatures (defined as hCN-SIG to denote extraction from hematological cancer data). **c**) A heatmap of hCN-SIG in the validation WGS according to chromothripsis. (Purple = containing chromothripsis; grey = lacking chromothripsis).

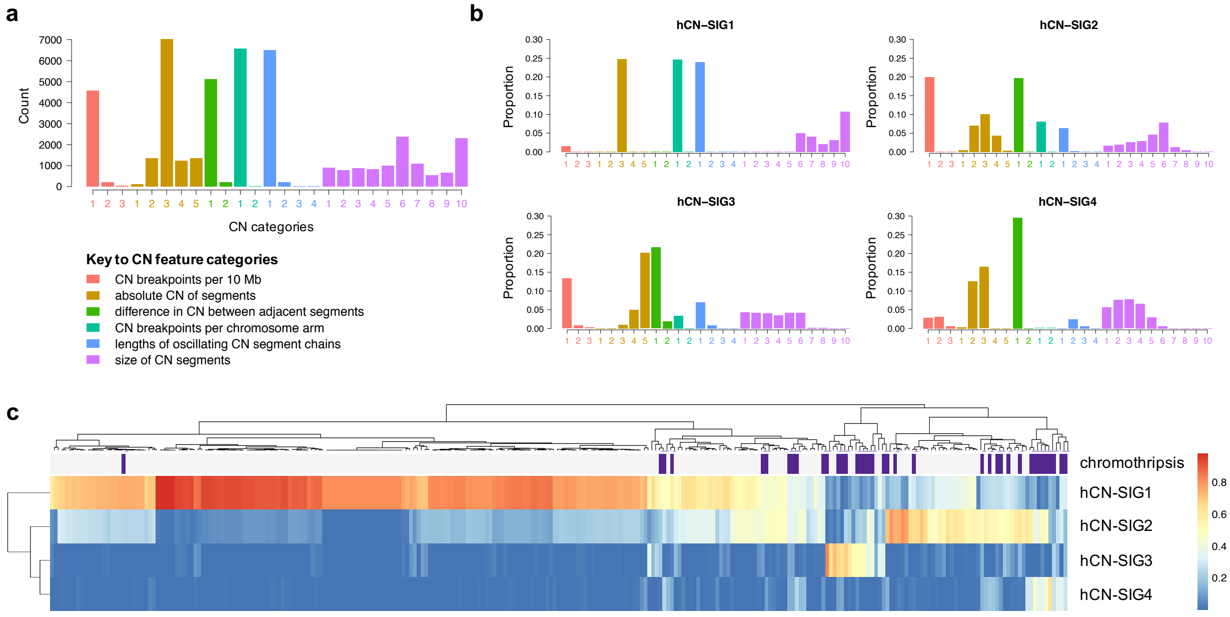

Supplementary Figure 9. Survival according to copy number (CN) signatures in newly diagnosed multiple myeloma closely resembles survival according to the presence or absence of chromothripsis. a) Progression-free survival (PFS) probability in the CoMMpass dataset according to the presence or absence of chromothripsis, and according to a high or low CN-prediction score for chromothripsis (CN-pred). **b**) Overall survival (OS) probability in the CoMMpass dataset according to the presence or absence of chromothripsis, and according to a high or low CN-pred score. (Chromothripsis; presence = green, absence = red, CN-pred; high = purple, low = blue, all p-values were generated according to the log rank test).

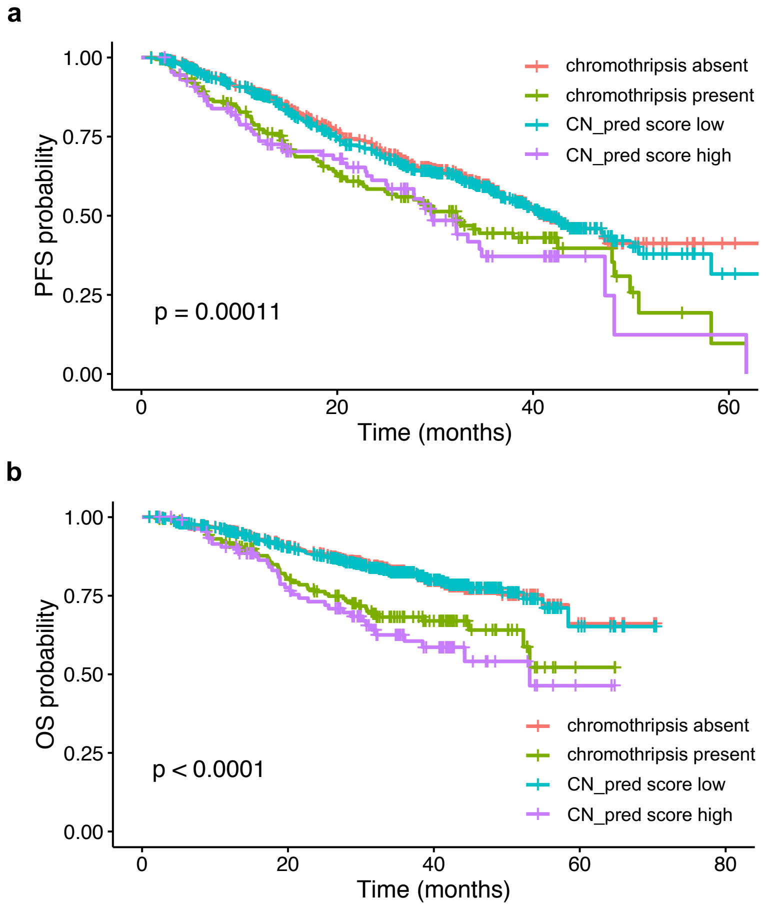

Supplementary Figure 10. Survival according to copy number (CN) signatures in newly diagnosed multiple myeloma is independent of treatment. Multivariate analysis of the effect of the copy number signatures prediction score (CN_pred) on (**a**) progression-free survival and (**b**) overall survival after correction for exposure to daratumumab (DARA), immunomodulatory agents (IMID), proteasome inhibitors (PI), International Staging Score (ISS), age, Eastern Cooperative Oncology Group (ECOG) score, APOBEC mutational activity and gain/amplification of 1q21. Multivariate analysis was performed by the Cox proportional hazards model with p-values according to a 2-sided Wald test. Data are presented as median values +/- 95% confidence interval, n = 752

a

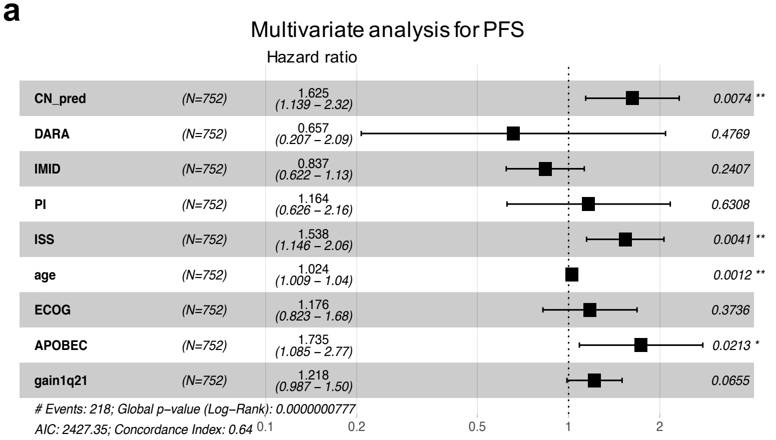

b

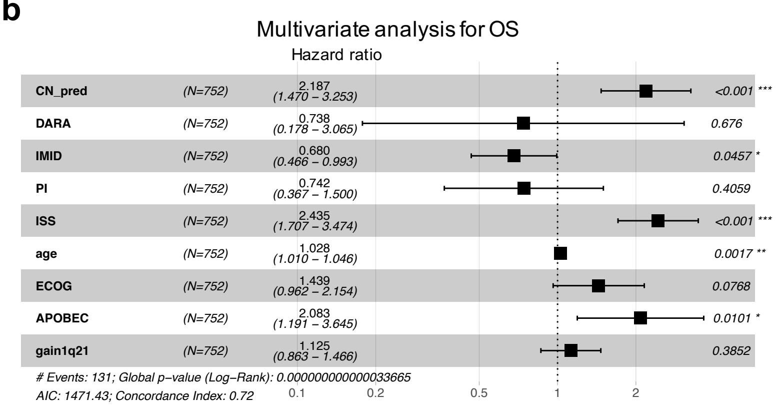

Supplementary Figure 11. Survival is shorter in patients having chromothripsis which was predicted by copy number (CN) signatures. Multivariate analysis of (**a**) progression-free survival (PFS) and (**d**) overall survival (OS) from whole genome sequencing after correction for age, International Staging Score (ISS), age, Eastern Cooperative Oncology Group (ECOG) score, gain/amplification of chromosome 1q21, and APOBEC mutational activity, according to discrepancy in chromothripsis prediction. Neg/neg = no chromothripsis by manual curation and chromothripsis not predicted by CN signatures, neg/pos = no chromothripsis but predicted by CN signatures, pos/neg = chromothripsis present but not predicted by CN signatures and pos/pos = chromothripsis present and predicted by CN signatures. Multivariate analysis was performed by the Cox proportional hazards model with p-values according to a 2-sided Wald test. Data are presented as median values +/- 95% confidence interval, n = 752.

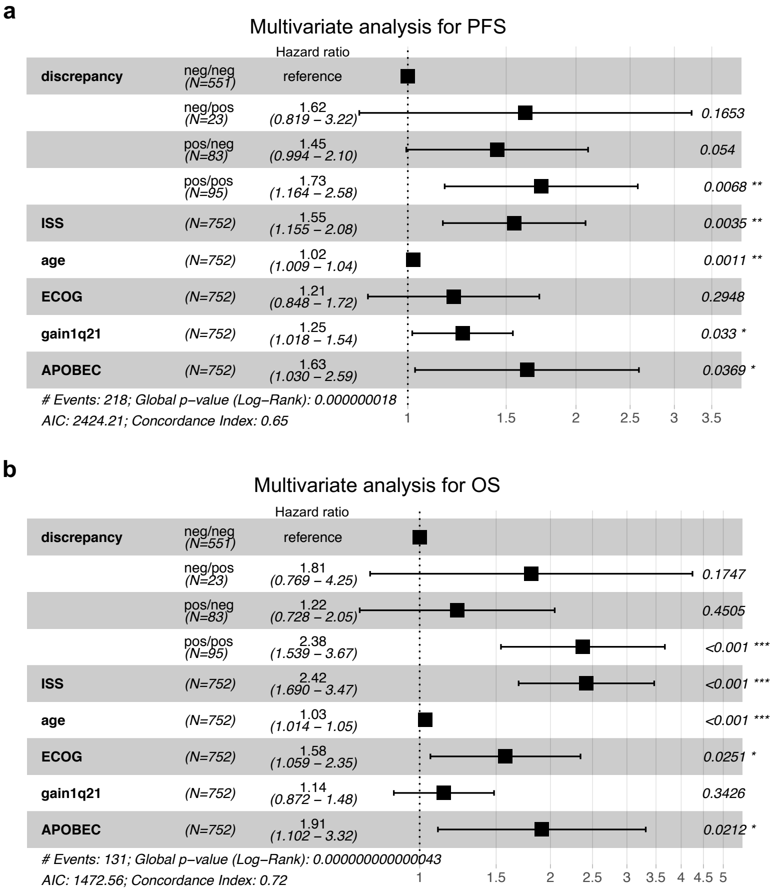

Supplementary Figure 12. Comparison of the area-under-the-curve (AUC) estimates for the prediction of chromothripsis between copy number (CN) signatures, the loss-of-heterozygosity (LOH) index and the genomic scar score (GSS) in WGS. Histograms presenting the distribution for each of (**a**) the LOH-index and (**b**) the GSS. Receiver operating curve (ROC) for the prediction of chromothripsis from the CoMMpass WGS data by (**c**) the LOH-index and (**d**) the GSS. Histograms presenting the results from bootstrap analysis comparing the difference in chromothripsis prediction between CN signatures and (**e**) the LOH index and (**f**), the GSS. (AUC: mean area-under-the-curve).

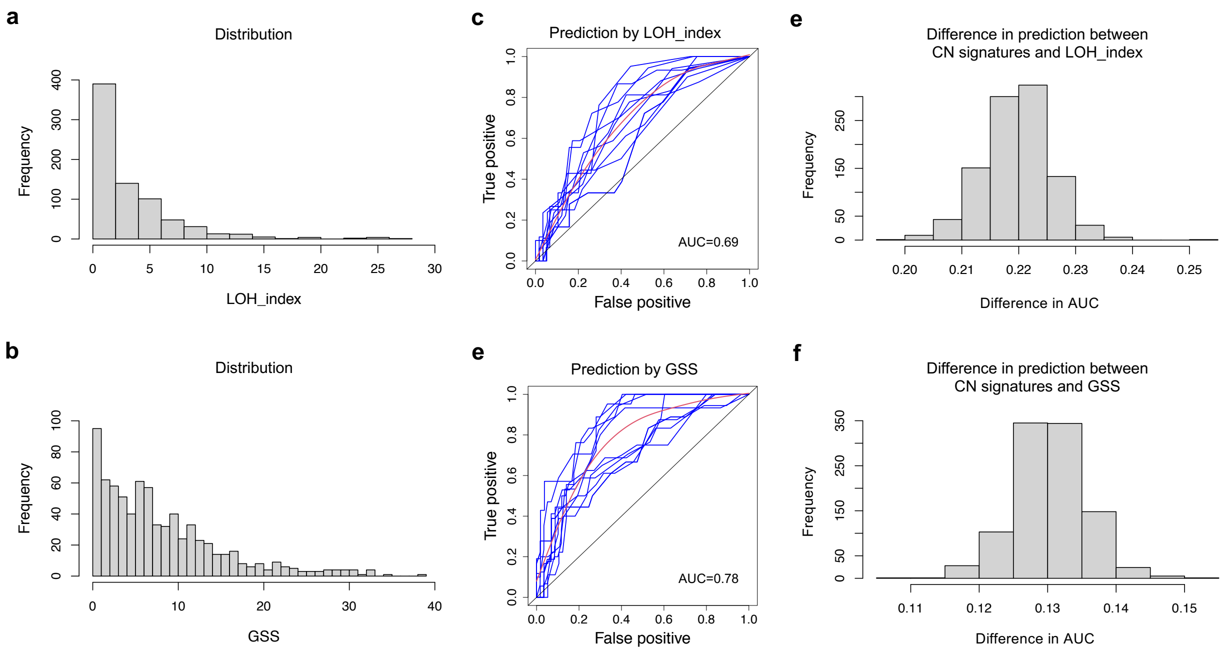

Supplementary Figure 13. Comparison of progression free and overall survival between copy number (CN) signatures, the loss-of-heterozygosity (LOH) index and the genomic scar score (GSS) in whole genome sequencing. Multivariate analysis of progression-free survival (PFS) from WGS after correction for age, International Staging Score (ISS), Eastern Cooperative Oncology Group (ECOG) score and APOBEC mutational activity according to (**a**) the LOH-index and (**b**) the GSS. Multivariate analysis of the effect on overall survival (OS) of (**c**) the LOH-index and (**d**) the GSS, after correction for the same factors. Multivariate analysis was performed by the Cox proportional hazards model with p-values according to a 2-sided Wald test. Data are presented as median values +/- 95% confidence interval, n = 752.

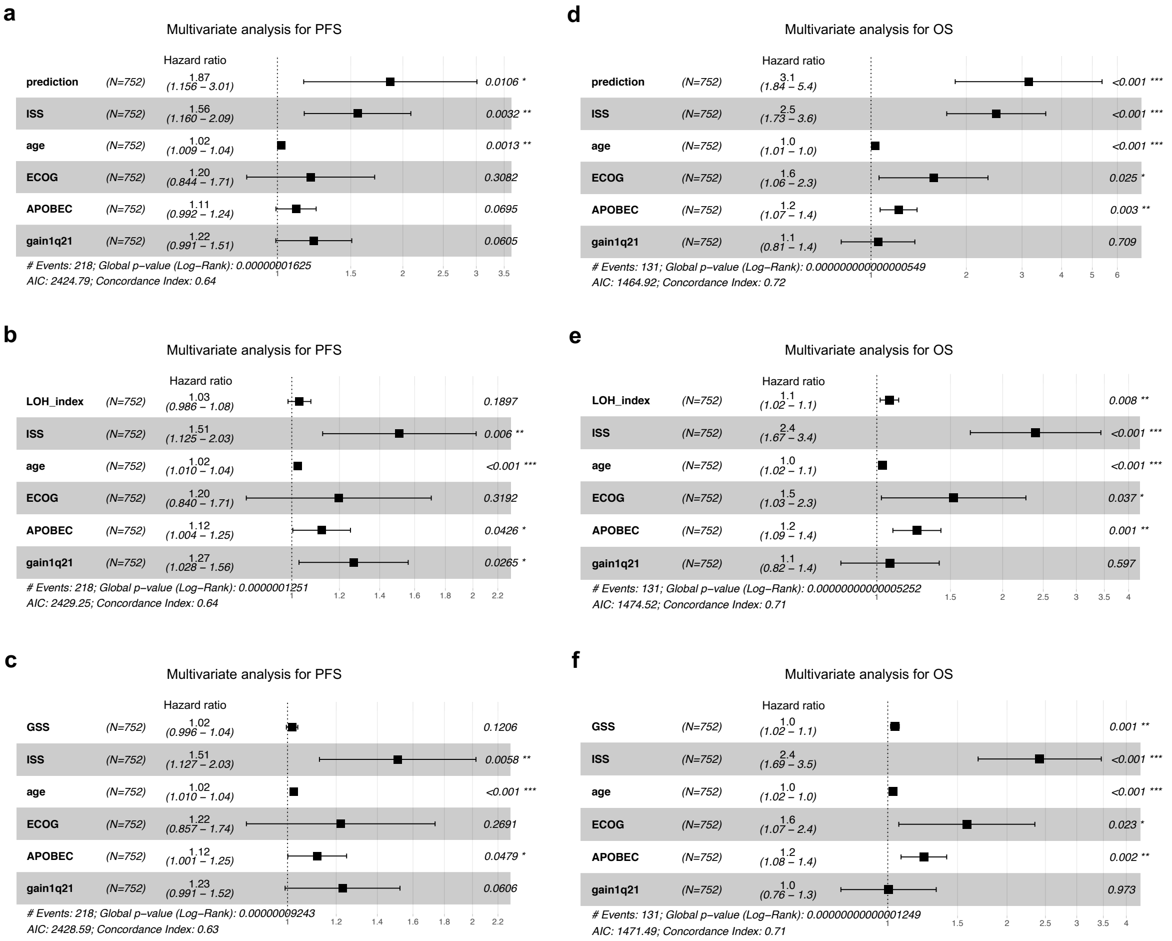

Supplementary Figure 14. Copy number (CN) feature matrix and CN signatures from the CoMMpass whole exome sequencing (WES) data. a) Using the CN feature limits defined in the CoMMpass WGS confirmed a similar CN feature matrix in the CoMMpass WES. **B**) *De novo* extraction without reference to WGS-derived CN signatures defined 5 CN signatures (defined as eCN-SIG to denote extraction from exome data). **c)** A heatmap of eCN signatures from WES data clustered by APOBEC mutational activity, *MAF/MAFB* translocations, t(4;14), gain / amplification of chromosome 1q21, biallelic *TP53* inactivation and chromothripsis. (Purple; chromothripsis present, grey; chromothripsis absent).

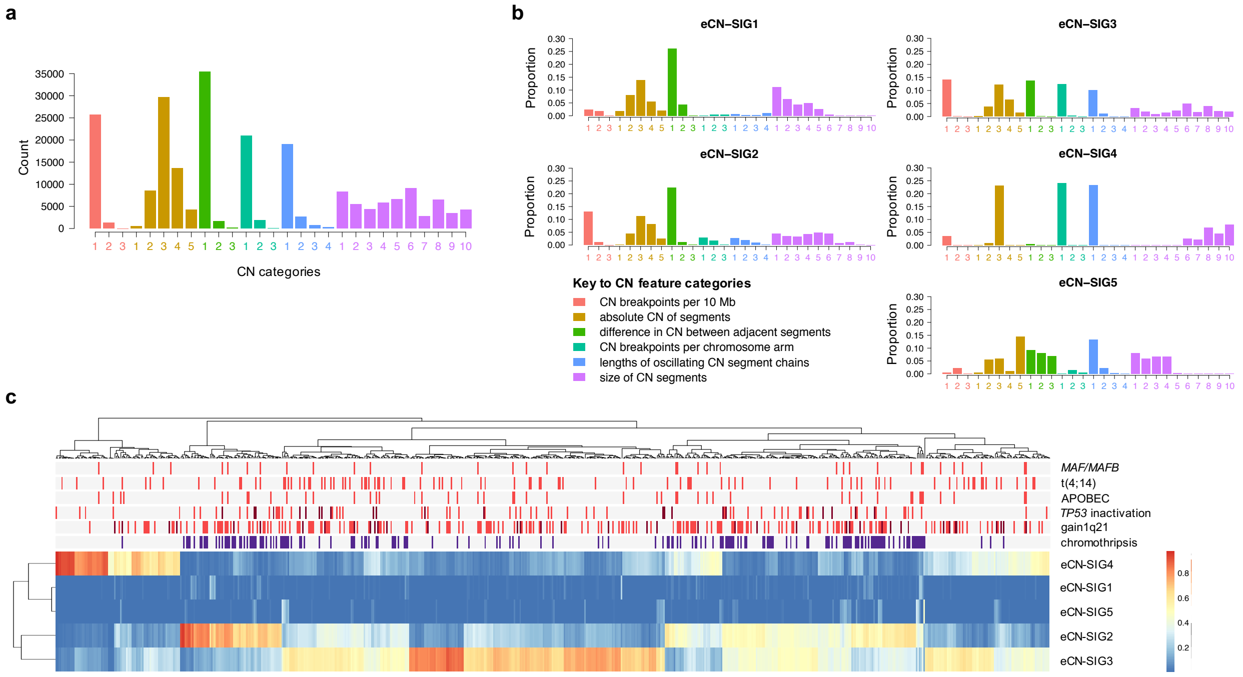

Supplementary Figure 15. Copy number (CN) signatures extracted from whole exome sequencing (WES) in newly diagnosed multiple myeloma are highly predictive of chromothripsis. Receiver operating curve (ROC) for the prediction of chromothripsis from CN signature analysis of CoMMpass WES data. (Blue lines; individual ROC from 10-fold cross validation, red line; mean of individual ROC, AUC: mean area-under-the-curve).

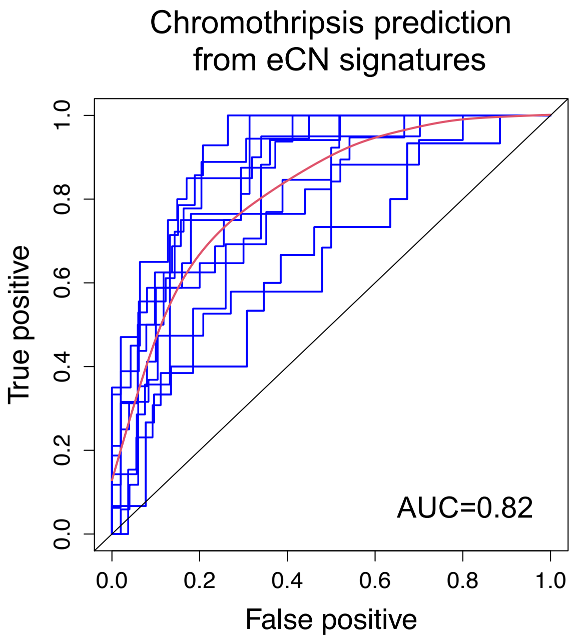

Supplementary Tables

Supplementary Table 1. Summary clinical details of patients from the CoMMpass dataset. ECOG = Eastern Cooperative Oncology Group; ISS = International Staging Score; IQR = interquartile range; m = months; n = number; PFS = progression-free survival; OS = overall survival.

Clinical variable	Value
Patients (n)	752
Gender (female, %)	39.9
Age (years, median, IQR)	64 (57 - 71)
ISS stage III (%)	26.5
ECOG status ≥2 (%)	17.3
PFS follow-up (median, IQR, m)	22.7 (11.3-35.2)
OS follow-up (median, IQR, m)	30.7 (15.9-41.0)

Supplementary Table 2. Twenty-eight copy number (CN) categories across 6 CN features defined from newly-diagnosed multiple myeloma patients in the CoMMpass dataset.

CN feature type	Category code	Count limit
breakpoints per 10 Mb	1	3
breakpoints per 10 Mb	2	6
breakpoints per 10 Mb	3	31
absolute CN	1	0
absolute CN	2	1
absolute CN	3	2
absolute CN	4	3
absolute CN	5	9
CN change point	1	1
CN change point	2	3
CN change point	3	8
breakpoints per chr arm	1	5
breakpoints per chr arm	2	17
breakpoints per chr arm	3	60
length of oscillating CN	1	1
length of oscillating CN	2	4
length of oscillating CN	3	9
length of oscillating CN	4	38
CN segment size	1	165300
CN segment size	2	502740
CN segment size	3	1588400
CN segment size	4	7113100
CN segment size	5	21801700
CN segment size	6	53962800
CN segment size	7	67587881
CN segment size	8	105333496
CN segment size	9	142322700
CN segment size	10	249137896

Supplementary Table 3. Copy number (CN) category contributions defining 5 CN signatures (CN-SIG) extracted from newly-diagnosed multiple myeloma patients in the CoMMpass dataset.

CN feature type	Category code	CN-SIG1	CN-SIG2	CN-SIG3	CN-SIG4	CN-SIG5
breakpoints per 10 Mb	1	0.0782	0.1500	0.1504	0.1134	0.0658
breakpoints per 10 Mb	2	0.0000	0.0058	0.0005	0.0158	0.0172
breakpoints per 10 Mb	3	0.0000	0.0000	0.0000	0.0030	0.0100
absolute CN	1	0.0000	0.0000	0.0038	0.0022	0.0013
absolute CN	2	0.0024	0.0032	0.0929	0.0922	0.0101
absolute CN	3	0.1778	0.1018	0.1453	0.1398	0.0456
absolute CN	4	0.0507	0.1234	0.0001	0.0313	0.1008
absolute CN	5	0.0044	0.0254	0.0000	0.0000	0.1117
CN change point	1	0.0356	0.1980	0.1388	0.2418	0.1516
CN change point	2	0.0003	0.0031	0.0009	0.0028	0.1065
CN change point	3	0.0000	0.0000	0.0000	0.0000	0.0052
breakpoints per chr arm	1	0.2123	0.0662	0.1239	0.0238	0.0004
breakpoints per chr arm	2	0.0000	0.0080	0.0013	0.0182	0.0140
breakpoints per chr arm	3	0.0000	0.0000	0.0000	0.0013	0.0040
length of oscillating CN	1	0.1994	0.0435	0.0942	0.0114	0.0478
length of oscillating CN	2	0.0006	0.0172	0.0083	0.0162	0.0278
length of oscillating CN	3	0.0000	0.0029	0.0003	0.0090	0.0099
length of oscillating CN	4	0.0000	0.0003	0.0000	0.0034	0.0001
CN segment size	1	0.0065	0.0447	0.0210	0.0338	0.0353
CN segment size	2	0.0077	0.0336	0.0175	0.0421	0.0529
CN segment size	3	0.0030	0.0259	0.0119	0.0483	0.0794
CN segment size	4	0.0011	0.0165	0.0147	0.0534	0.0615
CN segment size	5	0.0029	0.0420	0.0376	0.0495	0.0357
CN segment size	6	0.0261	0.0362	0.0362	0.0235	0.0047
CN segment size	7	0.0255	0.0192	0.0246	0.0116	0.0002
CN segment size	8	0.0618	0.0184	0.0384	0.0120	0.0002
CN segment size	9	0.0375	0.0048	0.0121	0.0000	0.0001
CN segment size	10	0.0659	0.0097	0.0252	0.0002	0.0002

Supplementary Table 4. Profiles of 10 structural variant (SV) signatures extracted from newly-diagnosed multiple myeloma patients in the CoMMpass dataset (del = deletion, tds = tandem duplication, inv = inversion, trans = translocation)

SV feature type	SV-SIG1	SV-SIG2	SV-SIG3	SV-SIG4	SV-SIG5	SV-SIG6	SV-SIG7	SV-SIG8	SV-SIG9	SV-SIG10
clustered_del_1-10Kb	0.0159	0.0090	0.0110	0.0000	0.0002	0.0000	0.0001	0.0000	0.0000	0.0000
clustered_del_10-100Kb	0.0076	0.0239	0.0241	0.0000	0.0047	0.0001	0.0001	0.0000	0.0001	0.0000
clustered_del_100Kb-1Mb	0.0279	0.0130	0.0274	0.0000	0.0004	0.0000	0.0000	0.0000	0.0000	0.0000
clustered_del_1Mb-10Mb	0.1805	0.0179	0.0359	0.0000	0.0001	0.0000	0.0001	0.0000	0.0000	0.0000
clustered_del_>10Mb	0.0170	0.1465	0.0151	0.0000	0.0002	0.0000	0.0001	0.0001	0.0000	0.0000
clustered_tds_1-10Kb	0.0085	0.0059	0.0132	0.0000	0.0001	0.0001	0.0003	0.0000	0.0000	0.0000
clustered_tds_10-100Kb	0.0050	0.0196	0.0213	0.0000	0.0004	0.0000	0.0001	0.0000	0.0000	0.0001
clustered_tds_100Kb-1Mb	0.0172	0.0114	0.0158	0.0000	0.0000	0.0000	0.0001	0.0000	0.0001	0.0000
clustered_tds_1Mb-10Mb	0.1481	0.0280	0.0385	0.0000	0.0002	0.0000	0.0000	0.0000	0.0000	0.0000
clustered_tds_>10Mb	0.0145	0.1462	0.0177	0.0000	0.0003	0.0000	0.0001	0.0000	0.0000	0.0000
clustered_inv_1-10Kb	0.0331	0.0464	0.0590	0.0001	0.0046	0.0001	0.0003	0.0000	0.0000	0.0000
clustered_inv_10-100Kb	0.0144	0.0322	0.0548	0.0000	0.0021	0.0000	0.0000	0.0000	0.0000	0.0001
clustered_inv_100Kb-1Mb	0.0518	0.0397	0.0371	0.0000	0.0002	0.0000	0.0000	0.0000	0.0000	0.0000
clustered_inv_1Mb-10Mb	0.3913	0.0597	0.0711	0.0000	0.0001	0.0000	0.0000	0.0000	0.0000	0.0000
clustered_inv_>10Mb	0.0219	0.3198	0.0270	0.0000	0.0002	0.0000	0.0001	0.0000	0.0000	0.0000
clustered_trans	0.0001	0.0001	0.4516	0.0000	0.0013	0.0000	0.0000	0.0000	0.0000	0.0000
non-clustered_del_1-10Kb	0.0009	0.0004	0.0001	0.0472	0.1708	0.0853	0.0005	0.0004	0.0000	0.0371
non-clustered_del_10-100Kb	0.0003	0.0005	0.0002	0.0911	0.5455	0.2572	0.0034	0.0011	0.0024	0.0108
non-clustered_del_100Kb-1Mb	0.0034	0.0005	0.0006	0.1462	0.2522	0.3141	0.0038	0.0021	0.0039	0.0007
non-clustered_del_1Mb-10Mb	0.0007	0.0001	0.0129	0.0812	0.0033	0.0158	0.0776	0.0003	0.0002	0.0009
non-clustered_del_>10Mb	0.0001	0.0023	0.0003	0.0566	0.0007	0.0044	0.1663	0.0027	0.0018	0.0226
non-clustered_tds_1-10Kb	0.0003	0.0001	0.0004	0.0112	0.0013	0.0618	0.0003	0.0457	0.0001	0.0016
non-clustered_tds_10-100Kb	0.0002	0.0005	0.0002	0.0002	0.0027	0.0554	0.0003	0.4015	0.0361	0.0009
non-clustered_tds_100Kb-1Mb	0.0002	0.0001	0.0002	0.0159	0.0003	0.0004	0.0005	0.0765	0.5640	0.0110
non-clustered_tds_1Mb-10Mb	0.0089	0.0002	0.0008	0.0210	0.0002	0.0002	0.0461	0.0002	0.0001	0.0752
non-clustered_tds_>10Mb	0.0005	0.0212	0.0074	0.0142	0.0004	0.0000	0.1173	0.0003	0.0033	0.0108
non-clustered_inv_1-10Kb	0.0003	0.0159	0.0091	0.0540	0.0012	0.0251	0.0302	0.0001	0.0001	0.0069
non-clustered_inv_10-100Kb	0.0008	0.0112	0.0010	0.0592	0.0012	0.0012	0.0086	0.0003	0.0004	0.0002
non-clustered_inv_100Kb-1Mb	0.0003	0.0007	0.0001	0.0908	0.0030	0.0002	0.0137	0.0004	0.0004	0.0015
non-clustered_inv_1Mb-10Mb	0.0227	0.0047	0.0167	0.0937	0.0004	0.0000	0.0926	0.0003	0.0002	0.0008
non-clustered_inv_>10Mb	0.0055	0.0219	0.0004	0.0896	0.0004	0.0002	0.3031	0.0008	0.0015	0.0027
non-clustered_trans	0.0004	0.0006	0.0287	0.1275	0.0014	0.1783	0.1341	0.4670	0.3849	0.8157

Supplementary Table 5. The sensitivity and specificity of chromothripsis prediction from copy-number (CN) signatures. The probability of chromothripsis was obtained by receiver operator curve analysis using all CN signatures from each cohort as the input. Highlighted in green is the chromothripsis prediction probability of 0.6 which was used in the prediction model. (CN-SIG = copy-number signature from CoMMpass WGS; hCN-SIG = hematological cancer copy-number signature; eCN-SIG = copy-number signature from CoMMpass exome sequencing.)

Prediction source	Probability	Sensitivity (%)	Specificity (%)
CN-SIG	**0.8**	36	98
	**0.7**	48	97
	**0.6**	53	96
	**0.5**	58	93
	**0.4**	67	92
	**0.3**	75	89
hCN-SIG	**0.8**	34	98
	**0.7**	41	98
	**0.6**	48	98
	**0.5**	62	97
	**0.4**	69	96
	**0.3**	72	96
eCN-SIG	**0.8**	8	97
	**0.7**	16	97
	**0.6**	24	95
	**0.5**	34	91
	**0.4**	47	88
	**0.3**	59	82

Supplementary Table 6. Summary clinical details of patients in the validation WGS dataset. IQR = interquartile range; n = number.

Clinical variable	Value
Patients (n)	269
Gender (female, %)	40.9
Age (years, median, IQR)	59 (49-68)
Cancer (n); multiple myeloma	34
chronic lymphocytic leukemia	92
chronic myeloid leukemia	29
acute myeloid leukemia	10
B-cell lymphoma	104

Supplementary Table 7. Profiles of the 4 copy number (CN) signatures (hCN-SIG) extracted from whole-genome sequencing of hematological patients in the validation dataset.

CN feature type	Category code	hCN-SIG1	hCN-SIG2	hCN-SIG3	hCN-SIG4
breakpoints per 10 Mb	1	0.0151	0.1999	0.1333	0.0281
breakpoints per 10 Mb	2	0.0000	0.0008	0.0080	0.0309
breakpoints per 10 Mb	3	0.0000	0.0000	0.0024	0.0056
absolute CN	1	0.0000	0.0047	0.0002	0.0030
absolute CN	2	0.0001	0.0695	0.0000	0.1261
absolute CN	3	0.2478	0.1008	0.0097	0.1640
absolute CN	4	0.0002	0.0431	0.0500	0.0003
absolute CN	5	0.0000	0.0030	0.2016	0.0008
CN change point	1	0.0001	0.1971	0.2160	0.2953
CN change point	2	0.0000	0.0000	0.0194	0.0001
breakpoints per chr arm	1	0.2465	0.0808	0.0334	0.0011
breakpoints per chr arm	2	0.0000	0.0000	0.0006	0.0011
length of oscillating CN	1	0.2402	0.0637	0.0698	0.0004
length of oscillating CN	2	0.0000	0.0024	0.0082	0.0246
length of oscillating CN	3	0.0000	0.0000	0.0002	0.0056
length of oscillating CN	4	0.0000	0.0000	0.0000	0.0006
CN segment size	1	0.0000	0.0172	0.0436	0.0568
CN segment size	2	0.0000	0.0189	0.0419	0.0768
CN segment size	3	0.0000	0.0261	0.0407	0.0773
CN segment size	4	0.0000	0.0288	0.0344	0.0659
CN segment size	5	0.0000	0.0464	0.0421	0.0298
CN segment size	6	0.0494	0.0788	0.0414	0.0055
CN segment size	7	0.0406	0.0125	0.0014	0.0000
CN segment size	8	0.0209	0.0052	0.0015	0.0001
CN segment size	9	0.0310	0.0000	0.0000	0.0000
CN segment size	10	0.1079	0.0001	0.0001	0.0000

Supplementary Table 8. Comparison between copy number signatures extracted from whole genome sequencing training and validation cohort, and those extracted from whole exome sequencing in newly diagnosed multiple myeloma. CN-SIG = copy-number signature from CoMMpass WGS; hCN-SIG = hematological cancer copy-number signature; eCN-SIG = copy-number signature from CoMMpass exome sequencing.

WGS CN signature	Validation CN signature	Validation cosine similarity	Exome CN signature	Exome cosine similarity
CN-SIG1	hCN-SIG1	0.956	eCN-SIG4	0.976
CN-SIG2	hCN-SIG2	0.920	eCN-SIG2	0.962
CN-SIG3	hCN-SIG2	0.923	eCN-SIG3	0.955
CN-SIG4	hCN-SIG4	0.943	eCN-SIG1	0.915
CN-SIG5	hCN-SIG3	0.850	eCN-SIG5	0.800

Supplementary Table 9. A matrix of copy number (CN) category contributions defining 5 CN signatures (eCN-SIG) extracted from whole-exome sequencing of newly-diagnosed multiple myeloma patients in the CoMMpass dataset.

CN feature type	Category code	eCN-SIG1	eCN-SIG2	eCN-SIG3	eCN-SIG4	eCN-SIG5
breakpoints per 10 Mb	1	0.0249	0.1291	0.1417	0.0350	0.0055
breakpoints per 10 Mb	2	0.0192	0.0108	0.0004	0.0000	0.0229
breakpoints per 10 Mb	3	0.0000	0.0000	0.0000	0.0000	0.0000
absolute CN	1	0.0192	0.0018	0.0021	0.0000	0.0040
absolute CN	2	0.0807	0.0443	0.0374	0.0086	0.0545
absolute CN	3	0.1380	0.1121	0.1214	0.2311	0.0593
absolute CN	4	0.0562	0.0805	0.0648	0.0002	0.0098
absolute CN	5	0.0210	0.0252	0.0150	0.0000	0.1445
CN change point	1	0.2606	0.2232	0.1371	0.0049	0.0917
CN change point	2	0.0446	0.0099	0.0013	0.0000	0.0813
CN change point	3	0.0001	0.0004	0.0000	0.0000	0.0699
breakpoints per chr arm	1	0.0004	0.0287	0.1236	0.2418	0.0007
breakpoints per chr arm	2	0.0053	0.0170	0.0027	0.0000	0.0142
breakpoints per chr arm	3	0.0047	0.0003	0.0000	0.0000	0.0058
length of oscillating CN	1	0.0060	0.0260	0.1013	0.2339	0.1324
length of oscillating CN	2	0.0022	0.0181	0.0104	0.0000	0.0222
length of oscillating CN	3	0.0025	0.0081	0.0002	0.0000	0.0024
length of oscillating CN	4	0.0110	0.0014	0.0000	0.0000	0.0001
CN segment size	1	0.1117	0.0445	0.0328	0.0003	0.0799
CN segment size	2	0.0657	0.0338	0.0177	0.0001	0.0598
CN segment size	3	0.0432	0.0325	0.0081	0.0008	0.0669
CN segment size	4	0.0495	0.0427	0.0140	0.0000	0.0675
CN segment size	5	0.0267	0.0481	0.0228	0.0000	0.0022
CN segment size	6	0.0057	0.0430	0.0497	0.0254	0.0003
CN segment size	7	0.0002	0.0076	0.0159	0.0226	0.0002
CN segment size	8	0.0003	0.0099	0.0392	0.0683	0.0004
CN segment size	9	0.0002	0.0013	0.0212	0.0460	0.0015
CN segment size	10	0.0002	0.0000	0.0192	0.0809	0.0003
