# Supplementary material for: Copy number signatures predict chromothripsis and clinical outcomes in newly diagnosed multiple myeloma
Source: Nat Commun. 2021 Aug 27;12:5172. doi: 10.1038/s41467-021-25469-8 (PMC8397708; doi:10.1038/s41467-021-25469-8)
Supplement: Supplementary file 3 — Description of Additional Supplementary Files [file 41467_2021_25469_MOESM3_ESM.pdf]

Description of Additional Supplementary Files

File Name: Supplementary Data 1
Description: applies copy number (CN) signature analysis to multiple myeloma. The optimal number of categories in each of 6 key CN features are defined by a mixed effect model, then de novo signature extraction is performed by the hierarchical Dirichlet process, producing a data frame detailing the relative proportional contribution from each CN signature.

File Name: Supplementary Data 2
Description: applies structural variant (SV) signature analysis to multiple myeloma. Using the size, type and clustering of SVs as input, de novo signature extraction is performed by the hierarchical Dirichlet process, producing a data frame detailing the relative proportional contribution from each SV signature.

File Name: Supplementary Data 3
Description: demonstrates how genomic signatures can be used to predict the presence of chromothripsis in multiple myeloma, defined by manual curation of copy number and structural variant data. Chromothripsis is predicted by estimating the average area-underthe-curve from receiver operating characteristic curves using 10-fold cross validation.

File Name: Supplementary Data 4
Description: demonstrates how copy number signatures are more accurate for the prediction of chromothripsis than an alternate copy number analysis tool. The genomic scar score (calculated using the R package scarHRD) sums 3 features (loss-of-heterozygosity, telomeric allelic imbalance, and number of large-scale transitions) to produce a final score. We estimated the average area-under-the-curve from receiver operating characteristic curves using 10-fold cross validation for each method, then calculated the difference in average AUC between the methods.
