# Supplementary material for: Copy number signatures predict chromothripsis and clinical outcomes in newly diagnosed multiple myeloma
Source: Nat Commun. 2021 Aug 27;12:5172. doi: 10.1038/s41467-021-25469-8 (PMC8397708; doi:10.1038/s41467-021-25469-8)
Supplement: Supplementary file 4 — Supplementary Data 1 [file 41467_2021_25469_MOESM4_ESM.html]

Applying copy number signature analysis in multiple myeloma


# Applying copy number signature analysis in multiple myeloma

#### Kylee Maclachlan, maclachk@mskcc.org

The following analysis workflow is a companion to the manuscript “Copy number signatures predict chromothripsis and clinical outcomes in newly diagnosed multiple myeloma” by Maclachlan et al.

Chromothripsis is a complex chromosomal shattering event associated with random rejoining, and is emerging as strong, independent adverse prognostic factor across multiple malignancies. Reliable detection of chromothripsis requires whole genome sequencing (WGS) and the integration of both structural variants (SVs) and copy number (CN) data.

Copy number (CN) signature analysis was described by Macintyre et al in Nature Genetics 2018 as a BRCAness surrogate. It takes WGS data and assesses 6 key copy number features. The optimal number of categories in each feature, which may differ between cancer types, is defined by a mixed effects model. De novo signature extraction is performed by the hierarchical Dirichlet process, producing a dataframe detailing the relative proportional contribution from each CN signature. Given the genome-wide distribution and complexity of chromothripsis in MM, here we apply CN signature analysis to MM.

In Supplementary Data 2 we apply SV signature analysis to MM. In Supplementary Data 3 we demonstrate how genomic signatures can be used to predict the presence of chromothripsis in MM, defined by manual curation of CN and SV data, estimating the average area-under-the-curve (AUC) from receiver operating characteristic (ROC) curves using 10-fold cross validation. In Supplementary Data 4 we demonstrate how CN signatures are more accurate than an alternate CN tool for the prediction of chromothripsis.

## Libraries

```
library(plyr)
library(dplyr)
library(hdp)
library(stringr)
library('GenomicFeatures')
library(BSgenome.Hsapiens.UCSC.hg19)
library(IRanges)
library("GenomicRanges")
library(RColorBrewer)
library(mclust)
library(pheatmap)
set.seed(1)
```

## Upload chromosome reference

```
snps = read.table("hg19.chrom_sizes.txt", header=F, sep="\t")
levels(snps$V2) <- c(levels(snps$V2), "chr23","chr24")
snps$V2[snps$V2 == "chrX"] <- "chr23"
snps$V2[snps$V2 == "chrY"] <- "chr24"
chr_list <- as.character(1:24) 

cyto <- list()
for  (i in (1:24))
{
  chr_num = i
  chr_filter <- paste("chr",chr_num,sep="")
  limit <- snps[snps$V2 == chr_filter,]
  post <- limit$V3
  x <- seq(from = 0, to = (post - 1000000), by = 1000000)
  p <- seq(from = 1000000, to = post, by = 1000000)
  alfa<- length(p)
  tail <-  c(p[alfa],post)
  y <- data.frame(x,p)
  y <- rbind(y,tail)
  y$chr <- rep(chr_filter,nrow(y))
  colnames(y)<-c("start","end","chr")  
  cyto[[chr_filter]] <- y
}
caryo <- do.call("rbind", cyto) # merge results from all chromosomes  
caryo$chr<-gsub("chr", "",caryo$chr)

gr1 = with(caryo, GRanges(chr, IRanges(start=start, end=end)))
```

## Upload reference for 10Mb chromosome bands

```
cyto_10mb <- list()
for  (i in c(1:22,"X"))
{
  chr_filter = i
  limit <- snps[snps$V1 == chr_filter,]
  post <- limit$V3
  x <- seq(from = 0, to = (post - 10000000), by = 10000000)
  p <- seq(from = 10000000, to = post, by = 10000000)
  alfa<- length(p)
  tail <-  c(p[alfa],post)
  y <- data.frame(x,p)
  y <- rbind(y,tail)
  y$chr <- rep(chr_filter,nrow(y))
  colnames(y)<-c("start","end","chr")  
  cyto_10mb[[chr_filter]] <- y
}
cyto_10mb_all <- do.call("rbind", cyto_10mb)
gr1_10mb = with(cyto_10mb_all, GRanges(chr, IRanges(start=start, end=end)))
```

## Upload CoMMpass copy number data

Limited to samples with good quality SV data as defined by manual inspection and curation.

```
# Final CN calls.
cnv_mmrf_sel<- read.delim("CoMMpass_CN_data.txt", stringsAsFactors = F)
length(unique(cnv_mmrf_sel$sample))
```

```
## [1] 752
```

```
head(cnv_mmrf_sel)
```

```
##           sample Chrom    start      end major minor
## 1 MMRF_1016_1_BM     1   571000   720500     1     0
## 2 MMRF_1016_1_BM     1   730700  9450900     2     1
## 3 MMRF_1016_1_BM     1  9456900  9820400     1     0
## 4 MMRF_1016_1_BM     1  9826900 16199000     2     1
## 5 MMRF_1016_1_BM     1 16204900 16299400     1     0
## 6 MMRF_1016_1_BM     1 16304400 33442559     2     1
```

## Quality adjustments

```
# Remove IgH, IgK and IgL loci to reduce artefacts due to VDJ-rearrangement / class-switch recombination
cnv_mmrf_sel$code_row<- 1:nrow(cnv_mmrf_sel)
igh_cnv <- cnv_mmrf_sel[cnv_mmrf_sel$Chrom == 14 & cnv_mmrf_sel$start >106032614 &  cnv_mmrf_sel$end< 108288051 | 
                      cnv_mmrf_sel$Chrom == 22 & cnv_mmrf_sel$start >21080474. &  cnv_mmrf_sel$end< 26065085 | 
                      cnv_mmrf_sel$Chrom == 2 & cnv_mmrf_sel$start >87090568 &  cnv_mmrf_sel$end< 93274235,]
cnv_mmrf_no_igh<- cnv_mmrf_sel[!cnv_mmrf_sel$code_row %in% igh_cnv$code_row ,]

# Remove chromosome X to avoid overestimation of deletion
cnv_mmrf_no_igh_no_x<- cnv_mmrf_no_igh[cnv_mmrf_no_igh$Chrom!="X",]

# Remove segments < 50Mb in size
cnv_mmrf_final<- cnv_mmrf_no_igh_no_x[(cnv_mmrf_no_igh_no_x$end-cnv_mmrf_no_igh_no_x$start)>50000,]

# Collapse adjacent segments with the same copy number
cnv_mmrf2<- list()
sample_list<- unique(cnv_mmrf_final$sample)
for(j in (1:length(sample_list)))
{
  cna_mmrf_single<- cnv_mmrf_final[cnv_mmrf_final$sample == sample_list[j],]
  sam_cnv_list<- list()
  chr_list<- unique(cna_mmrf_single$Chrom)
  for(i in (1:length(chr_list)))
  {
    cna_mmrf_single_chr<- cna_mmrf_single[cna_mmrf_single$Chrom == chr_list[i],]

    list_chr<- list()
    vec<- rle((paste(cna_mmrf_single_chr$major)))$length 
    for(w in (1:length(vec)))
    {
      if(w==1){
        int<- cna_mmrf_single_chr[1:vec[w],]
        cna_mmrf_single_row<- c(int$sample[1], int$Chrom[1], int$start[1], int$end[nrow(int)], int$major[1], max(int$minor))
      }else{
        int<- cna_mmrf_single_chr[(sum(vec[1:(w-1)])+1):sum(vec[1:(w)]),]
        cna_mmrf_single_row<- c(int$sample[1], int$Chrom[1], int$start[1], int$end[nrow(int)], int$major[1], max(int$minor))
      }
      list_chr[[w]]<- cna_mmrf_single_row
    }
    list_chr2<- do.call("rbind",list_chr)
    sam_cnv_list[[i]]<- list_chr2
  }
  sam_cnv_list2<- do.call("rbind", sam_cnv_list)
  cnv_mmrf2[[j]]<-  sam_cnv_list2
}
cnv_mmrf<- do.call("rbind", cnv_mmrf2)
cnv_mmrf<- as.data.frame.matrix(cnv_mmrf)
colnames(cnv_mmrf)<-c("sample","Chrom","start","end", "major","minor")
cnv_mmrf$sample<- as.character(as.character(cnv_mmrf$sample))
cnv_mmrf$Chrom<- as.character(as.character(cnv_mmrf$Chrom))
cnv_mmrf[,3:ncol(cnv_mmrf)]<- apply(cnv_mmrf[,3:ncol(cnv_mmrf)], 2, function(x){as.numeric(as.character(x))})
```

## Generate matrix of copy number features

```
mat_sig_cnv_final<- list()
max_10mb<- 100
max_copy_number<- max(cnv_mmrf$major)

# Create list for each copy number feature
count_10mb_all<-list()
size_all<- list()
count_jump_all<- list()
count_cnv_all<- list()
band_rate_all<-list()
osci_all<- list()
sample_list<- unique(cnv_mmrf$sample)

# Start loop for each sample
for(w in (1:length(sample_list)))
{
cnv_mmrf2<- cnv_mmrf[cnv_mmrf$sample ==sample_list[w],]

# Segment size
cnv_mmrf2$seg_size<- (cnv_mmrf2$end - cnv_mmrf2$start) 
size_all[[w]]<-cnv_mmrf2[,c("sample","seg_size")]

# Number of breaks per 10Mb segment
cnv_temp_brk<- cnv_mmrf2[,c(1,2,3,5,6,7)]
cnv_mmrf2_second<- cnv_mmrf2[,c(1,2,4,5,6,7)]

# Remove diploid whole chromosome regions
int_dipl<- as.data.frame.matrix(table(cnv_mmrf2_second$Chrom, cnv_mmrf2_second$major))
diploid_chr<- rownames(int_dipl[int_dipl$`2`==1 & rowSums(int_dipl)==1,])

# Remove second, so each break is only counted once
cnv_temp_brk<- cnv_mmrf2_second[! cnv_mmrf2_second$Chrom %in% diploid_chr,]

gr_cna_comm = with(cnv_temp_brk, GRanges(Chrom, IRanges(start=end, end=end)))
values(gr_cna_comm) <- DataFrame(sample = cnv_temp_brk$sample, major = cnv_temp_brk$major, minor= cnv_temp_brk$minor, seg_size= cnv_temp_brk$seg_size)
range_10mb <- merge(as.data.frame(gr_cna_comm),as.data.frame(gr1_10mb),by="seqnames",suffixes=c("A","B"))
range_dri_10mb <- range_10mb[with(range_10mb, startB <= startA & endB >= endA),]
count_brk_10mb<- as.data.frame(table(paste(range_dri_10mb$seqnames, range_dri_10mb$startB)))

# Use the reference for 10Mb without CNV breaks
options("scipen"=100, digits=10) 
cyto_10mb_all$Var1<- paste(cyto_10mb_all$chr, cyto_10mb_all$start)
count_10mb_file <-join(cyto_10mb_all, count_brk_10mb, by="Var1")
count_10mb_file[is.na(count_10mb_file)]<-0
count_10mb_file$sample<- sample_list[w]
count_10mb_df<- count_10mb_file[,c("sample","Freq")]
colnames(count_10mb_df)[2]<-"count"
count_10mb_all[[w]]<-count_10mb_df 

# Assess adjacent change in copy number (jump)
cnv_mmrf2_second$jump<- NA
summary_jump<- as.data.frame(table(cnv_mmrf2_second$Chrom))
cnv_mmrf2_second_jump<- cnv_mmrf2_second
if(length(unique(cnv_mmrf2_second_jump$Chrom))!=0){
chr_list_jump<- unique(cnv_mmrf2_second_jump$Chrom)
cnv_mmrf2_second_jump$jump<- NA
all_chr_jump<- list()
for(jj in chr_list_jump){
  cnv_mmrf2_second_jump_int<- cnv_mmrf2_second_jump[cnv_mmrf2_second_jump$Chrom == jj,]
  for(z in (1:nrow(cnv_mmrf2_second_jump_int)))
  {
    if(z==1){
      cnv_mmrf2_second_jump_int$jump[1]=NA
    }else{
      cnv_mmrf2_second_jump_int$jump[z]<- abs((cnv_mmrf2_second_jump_int$major[z]) - 
                                             (cnv_mmrf2_second_jump_int$major[z-1]))
    }
  }
  all_chr_jump[[jj]]<-cnv_mmrf2_second_jump_int
}
all_chr_jump2<- do.call("rbind", all_chr_jump)
}else{
  all_chr_jump2<- cnv_mmrf2_second[1,]
  all_chr_jump2$jump<-0
}
all_chr_jump2<- all_chr_jump2[! is.na(all_chr_jump2$jump),]
temp_jump<- all_chr_jump2[,c("sample","jump")]
colnames(temp_jump)<- c("sample","count")
count_jump_all[[w]]<-temp_jump 

# Count absolute copy number of each segment
count_cnv_final_df<- cnv_mmrf2[,c("sample","major")]
colnames(count_cnv_final_df)[2]<-"count"
count_cnv_all[[w]]<- count_cnv_final_df[,c("sample","count")]

# Count breakpoints per chromosome arm
chrom_arms<- read.delim("CentromerePosition_hg19.txt")
chrom_arms$chrom<- gsub("chr", "",chrom_arms$chrom)
cnv_mmrf2_second<- cnv_mmrf2[,c(1,2,4,5,6,7)]
cnv_temp_brk_arm <- cnv_mmrf2_second

if(nrow(cnv_temp_brk_arm)!=0){
gr_cna_comm = with(cnv_temp_brk_arm, GRanges(Chrom, IRanges(start=end, end=end)))
values(gr_cna_comm) <- DataFrame(sample = cnv_temp_brk_arm$sample, 
                                 major = cnv_temp_brk_arm$major, minor= cnv_temp_brk_arm$minor, seg_size= cnv_temp_brk_arm$seg_size)

gr_band = with(chrom_arms, GRanges(chrom, IRanges(start=chromStart, end=chromEnd)))

range_arm <- merge(as.data.frame(gr_cna_comm),as.data.frame(gr_band),by="seqnames",suffixes=c("A","B"))
range_arm$arm<- NA
range_arm$arm[range_arm$startA> range_arm$endB]<-"q_arm"
range_arm$arm[range_arm$startA< range_arm$startB]<-"p_arm"
range_arm$arm[range_arm$startB <= range_arm$startA & range_arm$endB >= range_arm$startA]<-"centro"

table(paste(range_arm$seqnames, range_arm$arm))
db_arm_counts<- as.data.frame(table(paste(range_arm$seqnames, range_arm$arm)))
}else{
  db_arm_counts<- matrix(c("13 q_arm", 0), nrow=1)
  db_arm_counts<- as.data.frame(db_arm_counts)
  colnames(db_arm_counts)<- c("Var1","Freq")
}

file_int_band<- as.data.frame(c(paste0(c(1:22), (" p_arm")), paste0(c(1:22), (" q_arm"))))
colnames(file_int_band)<-"Var1"
band_rate<- join(file_int_band, db_arm_counts, by="Var1")
band_rate[is.na(band_rate)]<-0
band_rate$sample<- sample_list[w]
colnames(band_rate)[2]<-"count"
band_rate_all[[w]]<-band_rate[,c("sample","count")]

# Assess oscillating copy number length 
out<-c()
chrs<-unique(cnv_mmrf2$Chrom)
cnv_mmrf2$tot<- cnv_mmrf2$major
oscCounts<-c()
for(c in chrs)
{
  currseg<-cnv_mmrf2[cnv_mmrf2$Chrom==c,"tot"]
  currseg<-round(as.numeric(currseg))
  
  if(length(currseg)>3)
  {
    prevval<-currseg[1]
    count=0
    for(j in 3:length(currseg))
    {
      if(j==length(currseg)){
        oscCounts<-rbind(oscCounts,c(c,count))
        count=0
      }else{
        if(abs(currseg[j]-prevval)<=1 & currseg[j]!=currseg[j-1])
        {
          count<-count+1
        }else{
          oscCounts<-rbind(oscCounts,c(c,count))
          count=0
        }
      }
        prevval<-currseg[j-1]
    }
  }else{
    oscCounts<- rbind(oscCounts,c(c,0))
  }
}

oscCounts_df<- as.data.frame(oscCounts)
oscCounts_df$sample<- sample_list[w]
oscCounts_df$V2<-as.numeric(as.character(oscCounts_df$V2))
osci_all[[w]]<-oscCounts_df
}

# End of CNV feature extraction loop.
```

## Use a mixed effect model (mclust) to define the optimal number of categories for each copy number feature

```
set.seed(999)

# mclust for segment size classification
size_all22<-do.call("rbind", size_all)
size_all22<- as.data.frame(size_all22)
size_all22$seg_size<- as.numeric(as.character(size_all22$seg_size))
size_all22_alt<- size_all22
myMclust_size <- Mclust(size_all22_alt$seg_size,G=2:10,verbose=FALSE)
size_all22_alt$size_code <- myMclust_size$classification
size_coordinate<- aggregate(seg_size ~ size_code, data = size_all22_alt, max)

# define absolute CNV state
count_cnv_all2<-do.call("rbind", count_cnv_all)
count_cnv_all2$cnv_count_code<- NA
count_cnv_all2$cnv_count_code[count_cnv_all2$count==0]<- 1
count_cnv_all2$cnv_count_code[count_cnv_all2$count==1]<- 2
count_cnv_all2$cnv_count_code[count_cnv_all2$count==2]<- 3
count_cnv_all2$cnv_count_code[count_cnv_all2$count==3]<- 4
count_cnv_all2$cnv_count_code[count_cnv_all2$count>=4]<- 5
count_cnv_coordinate<- aggregate(count ~ cnv_count_code, data = count_cnv_all2, max)

# mclust for CNV breakpoints per 10Mb
count_10mb_all2<-do.call("rbind", count_10mb_all)
count_10mb_all2<- as.data.frame(count_10mb_all2)
count_10mb_all2$count<- as.numeric(as.character(count_10mb_all2$count))
count_10mb_all2_alt<- count_10mb_all2[count_10mb_all2$count!=0,]
myMclust_10mb <- Mclust(count_10mb_all2_alt$count,G=4:4,verbose=FALSE)
count_10mb_all2_alt$mb_code <- myMclust_10mb$classification
count_cnv_coordinate_10mb <- aggregate(count ~ mb_code, data = count_10mb_all2_alt, max)

# mclust for jumps between adjacent segments 
count_jump_all2<-do.call("rbind", count_jump_all)
count_jump_all2<- as.data.frame(count_jump_all2)
count_jump_all2$count<- as.numeric(as.character(count_jump_all2$count))
count_jump_all2_alt<- count_jump_all2
myMclust_jump<- Mclust(count_jump_all2_alt$count,G=2:3,verbose=FALSE)
count_jump_all2_alt$cnv_count_code <- myMclust_jump$classification
jump_coordinate_10mb <- aggregate(count ~ cnv_count_code, data = count_jump_all2_alt, max)

# mclust for breakpoints per chromosome arm
band_rate_all2<-do.call("rbind", band_rate_all)
band_rate_all2<- as.data.frame(band_rate_all2)
band_rate_all2$Freq<- as.numeric(as.character(band_rate_all2$count))
band_rate_all2_alt<- band_rate_all2[band_rate_all2$count!=0,]
myMclust_band<- Mclust(band_rate_all2_alt$count,G=5,verbose=FALSE)
band_rate_all2_alt$band_code <- myMclust_band$classification
band_coordinate <- aggregate(count ~ band_code, data = band_rate_all2_alt, max)

# mclust for oscillation
osci_all2<-do.call("rbind", osci_all)
osci_all2<- as.data.frame(osci_all2[,c(3,2)])
colnames(osci_all2)[2]<-"count"
osci_all2$count<- as.numeric(as.character(osci_all2$count))
osci_all2_alt<- osci_all2
myMclust_osci<- Mclust(osci_all2_alt$count,G=4:5,verbose=FALSE) 
osci_all2_alt$osci_code <- myMclust_osci$classification
osci_coordinate <- aggregate(count ~ osci_code, data = osci_all2_alt, max)
```

## Create a copy number feature table for reference in future studies

```
count_cnv_coordinate_10mb$type<- "10_MB"
colnames(count_cnv_coordinate_10mb)<-c("code","count_limit","type")
count_cnv_coordinate$type<-"cnv_count"
colnames(count_cnv_coordinate)<-c("code","count_limit","type")
jump_coordinate_10mb$type<- "jump"
colnames(jump_coordinate_10mb)<-c("code","count_limit","type")
band_coordinate$type<-"band"
colnames(band_coordinate)<-c("code","count_limit","type")
osci_coordinate$type<-"osci"
colnames(osci_coordinate)<-c("code","count_limit","type")
size_coordinate$type<-"size"
colnames(size_coordinate)<-c("code","count_limit","type")

final_classification_value<- rbind.data.frame(count_cnv_coordinate_10mb,
                                              count_cnv_coordinate,
                                              jump_coordinate_10mb,
                                              band_coordinate,
                                              osci_coordinate,
                                              size_coordinate
                                              )

# This data is shown in Supplementary Table 2
```

```
final_classification_value
```

```
##    code count_limit      type
## 1     1           3     10_MB
## 2     3           6     10_MB
## 3     4          31     10_MB
## 4     1           0 cnv_count
## 5     2           1 cnv_count
## 6     3           2 cnv_count
## 7     4           3 cnv_count
## 8     5           9 cnv_count
## 9     1           1      jump
## 10    2           3      jump
## 11    3           8      jump
## 12    1           5      band
## 13    4          17      band
## 14    5          60      band
## 15    1           1      osci
## 16    3           4      osci
## 17    4           9      osci
## 18    5          38      osci
## 19    1      171900      size
## 20    2      615800      size
## 21    3     2178800      size
## 22    4     7348000      size
## 23    5    24320926      size
## 24    6    53631700      size
## 25    7    66240800      size
## 26    8   107971048      size
## 27    9   144264141      size
## 28   10   249137896      size
```

## Create from mClust output a copy number feature matrix to input to hdp

```
osci_tab<- as.data.frame.matrix(table(osci_all2_alt$sample, osci_all2_alt$osci_code))
colnames(osci_tab)<- paste0("osci_", colnames(osci_tab))
osci_tab$sample<- rownames(osci_tab)

band_tab<- as.data.frame.matrix(table(band_rate_all2_alt$sample, band_rate_all2_alt$band_code))
colnames(band_tab)<- paste0("band_", colnames(band_tab))
band_tab$sample<- rownames(band_tab)

jump_tab<- as.data.frame.matrix(table(count_jump_all2_alt$sample, count_jump_all2_alt$cnv_count_code))
colnames(jump_tab)<- paste0("jump_", colnames(jump_tab))
jump_tab$sample<- rownames(jump_tab)

# Jump category has a small number of missing values- need to input 0 
miss_jump_sam<- osci_tab$sample[!osci_tab$sample %in% jump_tab$sample]
miss_jmp_file<- cbind(rep(0, length(miss_jump_sam)), rep(0, length(miss_jump_sam)), 
                      rep(0, length(miss_jump_sam)), miss_jump_sam)
miss_jmp_file<- as.data.frame(miss_jmp_file)
colnames(miss_jmp_file)<-colnames(jump_tab)
jump_tab<- rbind.data.frame(jump_tab, miss_jmp_file)

mb_10_tab<- as.data.frame.matrix(table(count_10mb_all2_alt$sample, count_10mb_all2_alt$mb_code))
colnames(mb_10_tab)<- paste0("mb_10_", colnames(mb_10_tab))
mb_10_tab$sample<- rownames(mb_10_tab)

count_tab<- as.data.frame.matrix(table(count_cnv_all2$sample, count_cnv_all2$cnv_count_code))
colnames(count_tab)<- paste0("count_cnv_", colnames(count_tab))
count_tab$sample<- rownames(count_tab)

size_tab<- as.data.frame.matrix(table(size_all22_alt$sample, size_all22_alt$size_code))
colnames(size_tab)<- paste0("size_cnv_", colnames(size_tab))
size_tab$sample<- rownames(size_tab)

hdp_final<-Reduce(merge, list(mb_10_tab, count_tab, jump_tab, 
                             band_tab, osci_tab, size_tab))
head(hdp_final)
```

```
##           sample mb_10_1 mb_10_3 mb_10_4 count_cnv_1 count_cnv_2 count_cnv_3
## 1 MMRF_1016_1_BM      22       2       0           0          12          31
## 2 MMRF_1021_1_BM      17       0       1           0           7          29
## 3 MMRF_1029_1_BM      11       1       0           0           3          21
## 4 MMRF_1030_1_BM      17       0       0           0           3          22
## 5 MMRF_1031_1_BM      60       2       0           0          12          52
## 6 MMRF_1032_1_BM      67       4       0           0          24          68
##   count_cnv_4 count_cnv_5 jump_1 jump_2 jump_3 band_1 band_4 band_5 osci_1
## 1          11           0     32      0      0     23      2      0     19
## 2           9           0     23      0      0     24      2      0     22
## 3           7           0      9      0      0     22      0      0     21
## 4          11           1     15      0      0     25      0      0     24
## 5          39           6     87      0      0     24      7      0     20
## 6          33           5    108      0      0     24      6      1     15
##   osci_3 osci_4 osci_5 size_cnv_1 size_cnv_2 size_cnv_3 size_cnv_4 size_cnv_5
## 1      1      1      1          6          7          4          7          3
## 2      1      1      0          4          2          4          4          4
## 3      1      0      0          3          0          0          1          0
## 4      0      0      0          6          1          0          0          4
## 5      4      3      1         28         11          2         12         21
## 6      6      3      2         22         14         14         18         26
##   size_cnv_6 size_cnv_7 size_cnv_8 size_cnv_9 size_cnv_10
## 1          7          3          5          5           7
## 2          7          4          6          3           7
## 3          7          3          7          2           8
## 4          4          3          8          3           8
## 5         13          6         13          0           3
## 6         16          6         10          1           3
```

## Plot heatmap to vizualize copy number feature distribution

Check for extreme outliers which may indicate artefacts.

```
rownames(hdp_final)<- hdp_final$sample
hdp_final2<- hdp_final[,-1] # remnove column "sample"
hdp_final2[,1:ncol(hdp_final2)]<- apply(hdp_final2[,1:ncol(hdp_final2)], 2, function(x){as.numeric(as.character(x))})
pheatmap(t(hdp_final2), show_colnames = FALSE)
```

## Barplot of the CN feature distribution of the entire dataset

Create a color function for plotting CN barplots, to match the CN feature categories in Macintyre et al in Nature Genetics 2018. Select the correct number of colors for the number of CN categories extracted by Mclust, which may vary between cancer types and the type of sequencing data.

```
mat_final<- hdp_final2

n=30
gg_color_hue <- function(n) {
  hues = seq(15, 375, length = n + 1)
  hcl(h = hues, l = 65, c = 100)[1:n]
}
cols = gg_color_hue(n)

cnv_colors<- cols[c(1,5,10, 14,21, 24)]
cnv_colors_final<- c(rep(cnv_colors[1], 3), rep(cnv_colors[2], 5), rep(cnv_colors[3], 3), 
                     rep(cnv_colors[4], 3), rep(cnv_colors[5], 4), rep(cnv_colors[6], 10))

channel_names2 <- c("1", "2", "3", "1", "2", "3", "4", "5", "1", "2", "3", "1", "2", "3", "1", "2", "3", "4", "1", "2", "3", "4", "5", "6", "7", "8", "9", "10")

x <-barplot(colSums(mat_final), las=2,
        col=cnv_colors_final, border = NA, xaxt = "n", cex.axis = 1.15)
axis(1, at=x,  label=rep("",28), mgp= c(3,1,0.2))
mtext(1, at=x, text=c(channel_names2), col=cnv_colors_final, padj = 1.5, cex = 1.15)
```

## Vizualize the CN feature distribution in each sample

```
#pdf("CNV_PLOT_PROFILE_test.PDF")
# for(i in (1:nrow(mat_final))){
#   x <-barplot(as.numeric(mat_final[i,]), main = rownames(mat_final[i,]), las=2,
#           col=cnv_colors_final, border = NA, xaxt = "n", cex.axis = 1.15)
#   axis(1, at=x,  label=rep("",28), mgp= c(3,1,0.2))
#  mtext(1, at=x, text=c(channel_names2), col=cnv_colors_final, padj = 1.5, cex = 1.15)
#  }
```

## Prepare to run hdp (hierarchical dirichlet process) for de novo signature extraction

```
names_pts<- rownames(mat_final)
channel_names<- colnames(mat_final)
genomicData<- (mat_final) 
n<- ncol(genomicData)
 shape<- 1
 invscale<- 1
 hdp<- hdp_init(ppindex=0, #index of the parent DP for initial DP
                cpindex=1, #index of alphaa and alphab for initial DP
                hh=rep(1/n,n), #params for base distn (uniform Dirichlet)
                alphaa=shape,
                alphab=invscale)
 
 hdp<- hdp_adddp(hdp,
                 numdp=nrow(genomicData),
                 pp=1,
                 cp=1)
 
 hdp<- hdp_setdata(hdp= hdp,dpindex=1:nrow(genomicData)+1,data=genomicData)
 hdp<- dp_activate(hdp,1:(nrow(genomicData)+1),10)
```

## Run hdp

This runs 4 independent sampling chains. Note this step takes about 30 minutes for 752 low coverage long-insert WGS samples.

```
#chlist <- vector("list", 4)
# for (i in 1:4){
#   chlist[[i]] <- hdp_posterior(hdp,
#                                burnin=20000,
#                                n=100,
#                                space=50,
#                                cpiter=3,
#                                seed=i*1e4)
# }
# 
# mut_example_multi <- hdp_multi_chain(chlist)
# saveRDS(mut_example_multi, "mut_example_multi_4_chain.RDS")
```

## Extract CN signatures

Can adjust the cosine similarity or the minimum number of samples, each of which will change the number of signatures extracted. Visualize the overall number of data items attributed to each signature

```
mut_example_multi<- readRDS("mut_example_multi_0.85_10.RDS")
mut_example_multi_0.85_10 <- hdp_extract_components(mut_example_multi, cos.merge = 0.85, min.sample = 10) 
mut_example_multi<- mut_example_multi_0.85_10 # rename the best solution

par(mfrow=c(1,1), mar=c(5, 4, 4, 2))
plot_comp_size(mut_example_multi, bty="L")
```

## Visualize each extracted CN signature

```
mut_example_multi_plot <- mut_example_multi
posteriorMeans_plot<- t(comp_categ_distn(mut_example_multi_plot)[[1]])
rownames(posteriorMeans_plot)<-  channel_names2
plotnames<- c("offset", "CN-SIG1", "CN-SIG2", "CN-SIG3", "CN-SIG4", "CN-SIG5")

for  (i in (2:6))
{
x <- barplot(posteriorMeans_plot[,i], las=2,
        col=cnv_colors_final, border = NA, xaxt = "n",  cex.axis = 1.5, main= plotnames[i], ylim = c(0, 0.25), cex.main = 2)
axis(1, at=x,  label=rep("",28), mgp= c(3,1,0.2))
mtext(1, at=x, text=c(channel_names2), col=cnv_colors_final, padj = 1.5, cex = 1.5)
}
```

## Assess CN signature contribution in each sample

```
x<-((mut_example_multi@comp_dp_distn))
kk<- x[["mean"]]
rownames(kk)<-c("offset", rownames(genomicData))
colnames(kk)<-c("offset", "CN-SIG1", "CN-SIG2", "CN-SIG3", "CN-SIG4", "CN-SIG5")
head(kk)
```

```
##                        offset       CN-SIG1       CN-SIG2       CN-SIG3
## offset         0.026891638899 0.42945269158 0.18521828845 0.20204047791
## MMRF_1016_1_BM 0.001824644550 0.37861374408 0.08881516588 0.09925355450
## MMRF_1021_1_BM 0.002941988950 0.47676795580 0.09290055249 0.12820441989
## MMRF_1029_1_BM 0.003307086614 0.69527559055 0.12582677165 0.12488188976
## MMRF_1030_1_BM 0.001887096774 0.54354838710 0.29375806452 0.14254838710
## MMRF_1031_1_BM 0.006502347418 0.08781690141 0.60670774648 0.05129694836
##                      CN-SIG4        CN-SIG5
## offset         0.12624278465 0.030154118510
## MMRF_1016_1_BM 0.42988151659 0.001611374408
## MMRF_1021_1_BM 0.29429558011 0.004889502762
## MMRF_1029_1_BM 0.04899606299 0.001712598425
## MMRF_1030_1_BM 0.01704838710 0.001209677419
## MMRF_1031_1_BM 0.24602112676 0.001654929577
```

```
# This data is shown in Supplementary Table 3.
```
