# Supplementary material for: Copy number signatures predict chromothripsis and clinical outcomes in newly diagnosed multiple myeloma
Source: Nat Commun. 2021 Aug 27;12:5172. doi: 10.1038/s41467-021-25469-8 (PMC8397708; doi:10.1038/s41467-021-25469-8)
Supplement: Supplementary file 5 — Supplementary Data 2 [file 41467_2021_25469_MOESM5_ESM.html]

Applying structural variant signature analysis in multiple myeloma


# Applying structural variant signature analysis in multiple myeloma

#### Kylee Maclachlan, maclachk@mskcc.org

The following analysis workflow is a companion to the manuscript “Copy number signatures predict chromothripsis and clinical outcomes in newly diagnosed multiple myeloma” by Maclachlan et al.

Chromothripsis is a complex chromosomal shattering event associated with random rejoining, and is emerging as strong, independent adverse prognostic factor across multiple malignancies. Reliable detection of chromothripsis requires whole genome sequencing (WGS) and the integration of both structural variants (SVs) and copy number (CN) data.

In Supplementary Data 1 we apply copy number (CN) signature analysis to MM. Here we demonstrate SV signature analysis in MM; using whole genome sequencing (WGS) data to produce a dataframe detailing the relative proportional contribution from each signature. In Supplementary Data 3 we demonstrate how genomic signatures can be used to predict the presence of chromothripsis in MM, defined by manual curation of CN and SV data, estimating the average area-under-the-curve (AUC) from receiver operating characteristic (ROC) curves using 10-fold cross validation. In Supplementary Data 4 we demonstrate how CN signatures are more accurate than an alternate CN tool for the prediction of chromothripsis.

## Libraries

```
library(plyr)
library(dplyr)
library(hdp)
library(RColorBrewer)
```

## Upload CoMMpass SV file

```
SV <- read.delim("CoMMpass_SV_for_signatures.txt", stringsAsFactors = F)
head(SV)
```

```
##   chrom1     pos1 chrom2     pos2         sample SVTYPE   bkdist       cluster
## 1      1  9444547      1  9817522 MMRF_1016_1_BM    DEL   372975 non-clustered
## 2      1 16197410      1 16298377 MMRF_1016_1_BM    DEL   100967 non-clustered
## 3      1 47955015      1 47968592 MMRF_1016_1_BM    INV    13577 non-clustered
## 4      1 47967693      1 76432733 MMRF_1016_1_BM    DUP 28465040 non-clustered
## 5      1 51682697      1 77373540 MMRF_1016_1_BM    INV 25690843 non-clustered
## 6      1 74634385      1 75327028 MMRF_1016_1_BM    INV   692643     clustered
##                                       code       sv_pcawg    PCAWG_class
## 1   1_9444547_1_9817522_MMRF_1016_1_BM_DEL            DEL         single
## 2 1_16197410_1_16298377_MMRF_1016_1_BM_DEL            DEL         single
## 3 1_47955015_1_47968592_MMRF_1016_1_BM_INV chromothripsis chromothripsis
## 4 1_47967693_1_76432733_MMRF_1016_1_BM_DUP chromothripsis chromothripsis
## 5 1_51682697_1_77373540_MMRF_1016_1_BM_INV chromothripsis chromothripsis
## 6 1_74634385_1_75327028_MMRF_1016_1_BM_INV chromothripsis chromothripsis
##   chrom_involved      size sv_code                         sig
## 1                100Kb-1Mb     del non-clustered_del_100Kb-1Mb
## 2                100Kb-1Mb     del non-clustered_del_100Kb-1Mb
## 3        1_11_20  10-100Kb     inv  non-clustered_inv_10-100Kb
## 4        1_11_20     >10Mb     tds     non-clustered_tds_>10Mb
## 5        1_11_20     >10Mb     inv     non-clustered_inv_>10Mb
## 6        1_11_20 100Kb-1Mb     inv     clustered_inv_100Kb-1Mb
```

```
length(unique(SV$sample))
```

```
## [1] 746
```

```
# 6 of 752 samples used in defining CN signatures have no SV.
```

```
length(unique(SV$sig))
```

```
## [1] 32
```

## Select standardized SV feature classification to input to SV signature code

```
genomicData <- as.data.frame.matrix(table(SV$sample, SV$sig))
```

## Prepare to run hdp (hierarchical dirichlet process) for de novo signature extraction

```
genomicData<- genomicData[,c("clustered_del_1-10Kb", "clustered_del_10-100Kb", "clustered_del_100Kb-1Mb", "clustered_del_1Mb-10Mb", "clustered_del_>10Mb", "clustered_tds_1-10Kb","clustered_tds_10-100Kb", "clustered_tds_100Kb-1Mb", "clustered_tds_1Mb-10Mb", "clustered_tds_>10Mb", "clustered_inv_1-10Kb", "clustered_inv_10-100Kb", "clustered_inv_100Kb-1Mb",  "clustered_inv_1Mb-10Mb", "clustered_inv_>10Mb",  "clustered_trans", "non-clustered_del_1-10Kb",  "non-clustered_del_10-100Kb", "non-clustered_del_100Kb-1Mb", "non-clustered_del_1Mb-10Mb", "non-clustered_del_>10Mb", "non-clustered_tds_1-10Kb", "non-clustered_tds_10-100Kb", "non-clustered_tds_100Kb-1Mb", "non-clustered_tds_1Mb-10Mb", "non-clustered_tds_>10Mb", "non-clustered_inv_1-10Kb", "non-clustered_inv_10-100Kb", "non-clustered_inv_100Kb-1Mb", "non-clustered_inv_1Mb-10Mb", "non-clustered_inv_>10Mb", "non-clustered_trans")]
 n<- ncol(genomicData)
 shape<- 1
 invscale<- 1
 hdp<- hdp_init(ppindex=0, #index of the parent DP for initial DP
                cpindex=1, #index of alphaa and alphab for initial DP
                hh=rep(1/n,n), #params for base distn (uniform Dirichlet)
                alphaa=shape,
                alphab=invscale)
 
 hdp<- hdp_adddp(hdp,
                 numdp=nrow(genomicData),
                 pp=1,
                 cp=1)
 
 hdp<- hdp_setdata(hdp= hdp,dpindex=1:nrow(genomicData)+1,data=genomicData)
 hdp<- dp_activate(hdp,1:(nrow(genomicData)+1),10)
```

This runs 4 independent sampling chains. Note this step takes about 30 minutes for 746 low coverage long-insert WGS samples.

```
# chlist <- vector("list", 4)
# for (i in 1:4){
#   chlist[[i]] <- hdp_posterior(hdp,
#                                burnin=40000,
#                                n=50,
#                                space=50,
#                                cpiter=3,
#                                seed=i*1e4)
# }
# 
# mut_example_multi <- hdp_multi_chain(chlist)
# saveRDS(mut_example_multi,"SV_sigs_all_Delly_Manta_4ch_40000.RDS")
```

## Extract SV signatures

Can adjust the cosine similarity or the minimum number of samples, each of which will change the number of signatures extracted. Visualize the overall number of data items attributed to each signature

```
mut_example_multi<- readRDS("SV_sigs_all_Delly_Manta_4ch_40000.RDS")
mut_example_multi_0.85_10 <- hdp_extract_components(mut_example_multi, cos.merge = 0.85, min.sample =10) 
mut_example_multi<- mut_example_multi_0.85_10 # rename the best solution

par(mfrow=c(1,1), mar=c(5, 4, 4, 2))
plot_comp_size(mut_example_multi, bty="L")
```

## Visualize each extracted SV signature

```
mut_example_multi_plot <- mut_example_multi

class_sig <- c("clustered_del_1-10Kb", "clustered_del_10-100Kb", "clustered_del_100Kb-1Mb", "clustered_del_1Mb-10Mb", "clustered_del_>10Mb", "clustered_tds_1-10Kb","clustered_tds_10-100Kb", "clustered_tds_100Kb-1Mb", "clustered_tds_1Mb-10Mb", "clustered_tds_>10Mb", "clustered_inv_1-10Kb", "clustered_inv_10-100Kb", "clustered_inv_100Kb-1Mb",  "clustered_inv_1Mb-10Mb", "clustered_inv_>10Mb",  "clustered_trans", "non-clustered_del_1-10Kb",  "non-clustered_del_10-100Kb", "non-clustered_del_100Kb-1Mb", "non-clustered_del_1Mb-10Mb", "non-clustered_del_>10Mb", "non-clustered_tds_1-10Kb", "non-clustered_tds_10-100Kb", "non-clustered_tds_100Kb-1Mb", "non-clustered_tds_1Mb-10Mb", "non-clustered_tds_>10Mb", "non-clustered_inv_1-10Kb", "non-clustered_inv_10-100Kb", "non-clustered_inv_100Kb-1Mb", "non-clustered_inv_1Mb-10Mb", "non-clustered_inv_>10Mb", "non-clustered_trans")

group_factor <- factor(colnames(class_sig),  levels = class_sig)
posteriorMeans_plot<- t(comp_categ_distn(mut_example_multi_plot)[[1]])
rownames(posteriorMeans_plot)<-  class_sig

color_plot<- c(rep("firebrick2", 5), rep("green4", 5),rep("dodgerblue2", 5),"darkgrey",
               rep("firebrick2", 5),rep("green4", 5),rep("dodgerblue2", 5),"darkgrey")

plotlabels <- c("  1-10kb  ", "   10-100kb ", "100kb-1Mb ", "1-10Mb  ", ">10Mb  ", "  1-10kb  ", "   10-100kb ", "100kb-1Mb ", "1-10Mb  ", ">10Mb  ", "  1-10kb  ", "   10-100kb ", "100kb-1Mb ", "1-10Mb  ", ">10Mb  ", "   ", "  1-10kb  ", "   10-100kb ", "100kb-1Mb ", "1-10Mb  ", ">10Mb  ", "  1-10kb  ", "   10-100kb ", "100kb-1Mb ", "1-10Mb  ", ">10Mb  ", "  1-10kb  ", "   10-100kb ", "100kb-1Mb ", "1-10Mb  ", ">10Mb  ",  "   ")

## Rearrange extracted SV signatures to have those with clustered features as SIGS 1-3, the rest as SIGS 4-10, and exclude the offset signature

posteriorMeans_plot <- posteriorMeans_plot[, c(11,7,5,2,9,6,4,10,8,3)]
plotnames<- c("SV-SIG1", "SV-SIG2", "SV-SIG3", "SV-SIG4", "SV-SIG5", "SV-SIG6", "SV-SIG7", "SV-SIG8", "SV-SIG9", "SV-SIG10")

for  (i in (1:10)) 
{
x <- barplot(posteriorMeans_plot[,i], las=2,
        col=color_plot, border = NA, xaxt = "n",  cex.axis = 1.5, main= plotnames[i], ylim = c(0, 1.0), cex.main = 2)
axis(1, at=x,  label=rep("",32), mgp= c(3,0.8,0.1))
mtext(1, at=x, text=c(plotlabels), col=color_plot, adj = 1.18, padj = 0.5, cex = 0.9, las = 2)
}
```
