# Supplementary material for: Copy number signatures predict chromothripsis and clinical outcomes in newly diagnosed multiple myeloma
Source: Nat Commun. 2021 Aug 27;12:5172. doi: 10.1038/s41467-021-25469-8 (PMC8397708; doi:10.1038/s41467-021-25469-8)
Supplement: Supplementary file 6 — Supplementary Data 3 [file 41467_2021_25469_MOESM6_ESM.html]

Testing the accuracy of SV and CN signatures for chromothripsis prediction


# Testing the accuracy of SV and CN signatures for chromothripsis prediction

#### Kylee Maclachlan, maclachk@mskcc.org

The following analysis workflow is a companion to the manuscript “Copy number signatures predict chromothripsis and clinical outcomes in newly diagnosed multiple myeloma” by Maclachlan et al.

Chromothripsis is a complex chromosomal shattering event associated with random rejoining, and is emerging as strong, independent adverse prognostic factor across multiple malignancies. Reliable detection of chromothripsis requires whole genome sequencing (WGS) and the integration of both structural variants (SVs) and copy number (CN) data.

CN signature analysis (analytical code in Supplementary Data 1) and SV signature analysis (code in Supplementary Data 2) take whole genome sequencing (WGS) data (or whole exome sequencing for CN signatures) and produce a dataframe detailing the relative proportional contribution from each signature.

Here we demonstrate how genomic signature analysis can be used to predict for the presence of chromothripsis by estimating the average area-under-the-curve (AUC) from receiver operating characteristic (ROC) curves using 10-fold cross validation.

In Supplementary Data 4 we demonstrate how CN signatures are more accurate than an alternate CN tool for the prediction of chromothripsis.

## Libraries

```
library(plyr)
library(dplyr)
library('pROC')
```

## Load chromothripsis calls and signature results

```
WGS_ALL_SIGS <- read.delim("WGS_ALL_SIGS.txt")
head(WGS_ALL_SIGS)
```

```
##           sample CN_SIG1 CN_SIG2 CN_SIG3 CN_SIG4  CN_SIG5 chromothripsis_code
## 1 MMRF_1016_1_BM 0.37861 0.08882 0.09925 0.42988 0.001611                   1
## 2 MMRF_1021_1_BM 0.47677 0.09290 0.12820 0.29430 0.004890                   0
## 3 MMRF_1029_1_BM 0.69528 0.12583 0.12488 0.04900 0.001713                   0
## 4 MMRF_1030_1_BM 0.54355 0.29376 0.14255 0.01705 0.001210                   0
## 5 MMRF_1031_1_BM 0.08782 0.60671 0.05130 0.24602 0.001655                   0
## 6 MMRF_1032_1_BM 0.06403 0.32360 0.05881 0.53281 0.006058                   1
##   SV_SIG1 SV_SIG2 SV_SIG3   SV_SIG4 SV_SIG5   SV_SIG6  SV_SIG7  SV_SIG8
## 1 0.26567 0.06450 0.31108 0.0783333 0.22058 0.0015000 0.001833 0.013750
## 2 0.17114 0.12795 0.16159 0.0004545 0.37091 0.0009091 0.005455 0.145227
## 3 0.52286 0.25071 0.10071 0.0007143 0.06357 0.0035714 0.015714 0.006429
## 4 0.56750 0.19600 0.05100 0.0005000 0.04200 0.0005000 0.001500 0.045000
## 5 0.07365 0.02750 0.05058 0.0003846 0.80635 0.0005769 0.001154 0.032692
## 6 0.18340 0.04263 0.16231 0.0939744 0.02891 0.3190385 0.001538 0.009231
##    SV_SIG9  SV_SIG10
## 1 0.010667 0.0005833
## 2 0.012273 0.0000000
## 3 0.027857 0.0000000
## 4 0.020000 0.0000000
## 5 0.004808 0.0000000
## 6 0.016154 0.0015385
```

## Predict chromothripsis from SV and CN signatures together, using using AUC estimation and 10-fold cross validation.

```
AUCV = NULL
ROCVSENS = NULL
ROCVSPENS = NULL
RUNV =NULL

Len = length(WGS_ALL_SIGS[,1]) 
SS = 1:Len
start=1
end=75 # if the dataset contains 752 samples, then 10x cross-fold validation requires 75 for this value
num_add<- 75 # and again here

for (i in 1:10){ 
  
  kk = SS[-(start:end)]
  start=start + num_add 
  end=end + num_add
  
  aacov1 = data.frame(chromth=WGS_ALL_SIGS$chromothripsis_code[kk],
                      factor1 = (WGS_ALL_SIGS$SV_SIG1)[kk],
                      factor2 = (WGS_ALL_SIGS$SV_SIG2)[kk],
                      factor3 = (WGS_ALL_SIGS$SV_SIG3)[kk],
                      factor4 = (WGS_ALL_SIGS$SV_SIG4)[kk],
                      factor5 = (WGS_ALL_SIGS$SV_SIG5)[kk],
                      factor6 = (WGS_ALL_SIGS$SV_SIG6)[kk],
                      factor7 = (WGS_ALL_SIGS$SV_SIG7)[kk],
                      factor8 = (WGS_ALL_SIGS$SV_SIG8)[kk],
                      factor9 = (WGS_ALL_SIGS$SV_SIG9)[kk],
                      factor10 = (WGS_ALL_SIGS$SV_SIG10)[kk],
                      factor12 = (WGS_ALL_SIGS$CN_SIG1)[kk],
                      factor13 = (WGS_ALL_SIGS$CN_SIG2)[kk],
                      factor14 = (WGS_ALL_SIGS$CN_SIG3)[kk],
                      factor15 = (WGS_ALL_SIGS$CN_SIG4)[kk],
                      factor16 = (WGS_ALL_SIGS$CN_SIG5)[kk]
  )
  
  aacov = glm(chromth~.,data = aacov1,family='binomial')

  aacov2 = data.frame(chromth=WGS_ALL_SIGS$chromothripsis_code[-kk],
                      factor1 = (WGS_ALL_SIGS$SV_SIG1)[-kk],
                      factor2 = (WGS_ALL_SIGS$SV_SIG2)[-kk],
                      factor3 = (WGS_ALL_SIGS$SV_SIG3)[-kk],
                      factor4 = (WGS_ALL_SIGS$SV_SIG4)[-kk],
                      factor5 = (WGS_ALL_SIGS$SV_SIG5)[-kk],
                      factor6 = (WGS_ALL_SIGS$SV_SIG6)[-kk],
                      factor7 = (WGS_ALL_SIGS$SV_SIG7)[-kk],
                      factor8 = (WGS_ALL_SIGS$SV_SIG8)[-kk],
                      factor9 = (WGS_ALL_SIGS$SV_SIG9)[-kk],
                      factor10 = (WGS_ALL_SIGS$SV_SIG10)[-kk],
                      factor12 = (WGS_ALL_SIGS$CN_SIG1)[-kk],
                      factor13 = (WGS_ALL_SIGS$CN_SIG2)[-kk],
                      factor14 = (WGS_ALL_SIGS$CN_SIG3)[-kk],
                      factor15 = (WGS_ALL_SIGS$CN_SIG4)[-kk],
                      factor16 = (WGS_ALL_SIGS$CN_SIG5)[-kk]
  )
  
  predpr <- predict(aacov,newdata=aacov2,type=c("response"))
  roccurve <- roc(aacov2$chromth,predpr)
  aa = auc(roccurve)
  aa = as.numeric(aa) 
  
  AUCV = c(AUCV,aa)
  ROCVSENS = c(ROCVSENS,roccurve$sensitivities)
  ROCVSPENS = c(ROCVSPENS,roccurve$specificities)
  RUNV =c(RUNV,rep(i,length(roccurve$specificities)))
}

aa<- mean(AUCV)
aa
```

```
## [1] 0.9601
```

```
# [1] 0.9601
```

## Vizualize the results by plotting the mean AUC from ROC analysis

```
Y = ROCVSENS
X1  = 1 - ROCVSPENS 

X11 = 1-roccurve$specificities 
Y11 = roccurve$sensitivities 
X11= sort(unique(X1))

par(mar = c(5, 5,3,2) +0.01)
fit <- smooth.spline(X1, Y, nknots = 10)
pred <- stats:::predict.smooth.spline(fit, X11)$y  

plot(X1[RUNV==1], Y[RUNV==1],lwd=1.7,type='l',col='blue',main=paste0('Chromothripsis from genomic sigs ', "AUC=",aa),
     ylab='True Positive',xlab='False Positive', cex.lab = 1.5, cex.axis = 1.3, mgp = c(3, 1,0))
for (kk in 1:10){
  points(X1[RUNV==kk], Y[RUNV==kk],type='l',col='blue',lwd=1.7)
}

lines(X11, pred, lwd = 2, col = 2) 
abline(a=0,b=1)
```
