# Supplementary material for: Copy number signatures predict chromothripsis and clinical outcomes in newly diagnosed multiple myeloma
Source: Nat Commun. 2021 Aug 27;12:5172. doi: 10.1038/s41467-021-25469-8 (PMC8397708; doi:10.1038/s41467-021-25469-8)
Supplement: Supplementary file 7 — Supplementary Data 4 [file 41467_2021_25469_MOESM7_ESM.html]

Comparing 2 copy number assessment methods for predicting chromothripsis


# Comparing 2 copy number assessment methods for predicting chromothripsis

#### Kylee Maclachlan, maclachk@mskcc.org

The following analysis workflow is a companion to the manuscript “Copy number signatures predict chromothripsis and clinical outcomes in newly diagnosed multiple myeloma” by Maclachlan et al.

Chromothripsis is a complex chromosomal shattering event associated with random rejoining, and is emerging as strong, independent adverse prognostic factor across multiple malignancies. Reliable detection of chromothripsis requires whole genome sequencing (WGS) and the integration of both structural variants (SVs) and copy number (CN) data.

CN signature analysis (Supplementary Data 1) and SV signature analysis (Supplementary Data 2) take whole genome sequencing (WGS) data (or whole exome sequencing for CN signatures) and produce a dataframe detailing the relative proportional contribution from each signature. This data can then be used to predict for the presence of chromothripsis, estimating the average area-under-the-curve (AUC) from receiver operating characteristic (ROC) curves using 10-fold cross validation (Supplementary Data 3).

Here we compare the prediction of chromothripsis from CN signatures with the prediction from another CN analysis tool; the genomic scar score (GSS) analysis (Samur et.al. JCO 2020). We obtained the GSS from WGS using the R package scarHRD, which sums 3 CN features (loss-of-heterozygosity, telomeric allelic imbalance, and number of large-scale transitions) to produce a final score.

To compare the accuracy of chromothripsis-prediction between the 2 methods, we first estimated the average AUC from receiver ROC curves via 10-fold cross validation for each method, then calculated the difference in average AUC between the methods. The standard deviation of the difference in AUCs was estimated by performing a bootstrap resampling; on each new bootstrap sample, we estimated difference in the average AUC between the methods using 10-fold cross-validation. This procedure was repeated 1000 times.

## Libraries

```
library(plyr)
library(dplyr)
library('pROC')
```

## Load data

```
# Output from CN signature  analysis 
WGS_CN_SIG <- read.delim("MMRF_752WGS_CN_SIG_results.txt")
dim(WGS_CN_SIG)
```

```
## [1] 752   7
```

```
head(WGS_CN_SIG)
```

```
##           sample CN_SIG1 CN_SIG2 CN_SIG3 CN_SIG4  CN_SIG5 chromothripsis_code
## 1 MMRF_1016_1_BM 0.37861 0.08882 0.09925 0.42988 0.001611                   1
## 2 MMRF_1021_1_BM 0.47677 0.09290 0.12820 0.29430 0.004890                   0
## 3 MMRF_1029_1_BM 0.69528 0.12583 0.12488 0.04900 0.001713                   0
## 4 MMRF_1030_1_BM 0.54355 0.29376 0.14255 0.01705 0.001210                   0
## 5 MMRF_1031_1_BM 0.08782 0.60671 0.05130 0.24602 0.001655                   0
## 6 MMRF_1032_1_BM 0.06403 0.32360 0.05881 0.53281 0.006058                   1
```

```
# Output from GSS analysis 
GSS <- read.delim("MMRF_WGS_genomic_scar_results.txt", stringsAsFactors = F)
dim(GSS)
```

```
## [1] 752   5
```

```
head(GSS)
```

```
##           sample HRD.LOH Telomeric.AI LST HRD.sum
## 1 MMRF_1016_1_BM       1            1   5       7
## 2 MMRF_1021_1_BM       2            5   3      10
## 3 MMRF_1029_1_BM       4            5   4      13
## 4 MMRF_1030_1_BM       2            2   2       6
## 5 MMRF_1031_1_BM       6            8   6      20
## 6 MMRF_1032_1_BM       5            7  11      23
```

```
datafr_genomes <- left_join(GSS, WGS_CN_SIG, by = "sample")
```

## Vizualize distribution of GSS scores

```
hist(datafr_genomes$HRD.sum, breaks =  40)
```

## Comparison of chromothripsis prediction methods

Define a function for chromothripsis prediction from CN signatures, using using AUC estimation and 10-fold cross validation.

```
my_method_AUC = function(datafr_genomes){
AUCV = NULL
ROCVSENS = NULL
ROCVSPENS = NULL
RUNV =NULL

Len = length(datafr_genomes[,1]) 
SS = 1:Len
start=1
end= 75 # if the dataset contains 752 samples, then 10x cross-fold validation requires 75 for this value
num_add<- 75 # and again here

for (i in 1:10){ 
  
  kk = SS[-(start:end)]
  start=start + num_add 
  end=end + num_add

  aacov1 = data.frame(chromth=datafr_genomes$chromothripsis_code[kk],
                      CN_SIG1 = (datafr_genomes$CN_SIG1)[kk],
                      CN_SIG2 = (datafr_genomes$CN_SIG2)[kk],
                      CN_SIG3 = (datafr_genomes$CN_SIG3)[kk],
                      CN_SIG4 = (datafr_genomes$CN_SIG4)[kk],
                      CN_SIG5 = (datafr_genomes$CN_SIG5)[kk]
  )
  
  aacov = glm(chromth~.,data = aacov1,family='binomial')

  aacov2 = data.frame(chromth=datafr_genomes$chromothripsis_code[-kk],
                      CN_SIG1 = (datafr_genomes$CN_SIG1)[-kk],
                      CN_SIG2 = (datafr_genomes$CN_SIG2)[-kk],
                      CN_SIG3 = (datafr_genomes$CN_SIG3)[-kk],
                      CN_SIG4 = (datafr_genomes$CN_SIG4)[-kk],
                      CN_SIG5 = (datafr_genomes$CN_SIG5)[-kk]

  )

  predpr <- predict(aacov,newdata=aacov2,type=c("response"))
  roccurve <- roc(aacov2$chromth,predpr)
  aa = auc(roccurve)  
  aa = as.numeric(aa) 
  
  AUCV = c(AUCV,aa)
  ROCVSENS = c(ROCVSENS,roccurve$sensitivities)
  ROCVSPENS = c(ROCVSPENS,roccurve$specificities)
  RUNV =c(RUNV,rep(i,length(roccurve$specificities)))
}

aa<- mean(AUCV)
aa
return (aa)
}

my_method_AUC(datafr_genomes)
```

```
## [1] 0.9031
```

Define a function for chromothripsis prediction from the CN features of the genomic scar score, using AUC estimation and 10-fold cross validation.

```
other_method_AUC = function(datafr_genomes){

AUCV = NULL
ROCVSENS = NULL
ROCVSPENS = NULL
RUNV =NULL

Len = length(datafr_genomes[,1]) 
SS = 1:Len
start=1
end= 75 # if the dataset contains 752 samples, then 10x cross-fold validation requires 75 for this value
num_add<- 75 # and again here

for (i in 1:10){ 
  
  kk = SS[-(start:end)]
  start=start + num_add 
  end=end + num_add
  
  aacov1 = data.frame(chromth=datafr_genomes$chromothripsis_code[kk],
                      CN_feature_1 = (datafr_genomes$HRD.sum)[kk]
  )
  
  aacov = glm(chromth~.,data = aacov1,family='binomial')

  aacov2 = data.frame(chromth=datafr_genomes$chromothripsis_code[-kk],
                      CN_feature_1 = (datafr_genomes$HRD.sum)[-kk]
  )
  
  predpr <- predict(aacov,newdata=aacov2,type=c("response"))
  roccurve <- roc(aacov2$chromth,predpr)
  aa = auc(roccurve)  
  aa = as.numeric(aa) 
  
  AUCV = c(AUCV,aa)
  ROCVSENS = c(ROCVSENS,roccurve$sensitivities)
  ROCVSPENS = c(ROCVSPENS,roccurve$specificities)
  RUNV =c(RUNV,rep(i,length(roccurve$specificities)))
}

aa<- mean(AUCV)
aa
return (aa)
}

other_method_AUC(datafr_genomes)
```

```
## [1] 0.7751
```

## Bootstrap analysis to test the reliability of chromothripsis prediction

```
AUC_my_method = NULL
AUC_other_method = NULL

L = length(datafr_genomes[,1])

# Outcome metric
Y <-  datafr_genomes$chromothripsis_code

# Predictors for each approach
COV_my_method = datafr_genomes
COV_other_method = datafr_genomes

# Difference between the 2 methods
deltaObs =  my_method_AUC(COV_my_method) - other_method_AUC(COV_other_method)

deltaV = NULL
for (j in 1:1000){
  kkk = sample(1:L, replace = T)
  Ys = Y[kkk]
  COV_my_method = datafr_genomes[kkk,]
  COV_other_method = datafr_genomes[kkk,]
  
  deltas=  my_method_AUC(COV_my_method) - other_method_AUC(COV_other_method)
  deltaV = c(deltaV,deltas)
}
```

# Difference in estimation accuracy between the 2 methods

```
mean(deltaV)
```

```
## [1] 0.1285
```

## Vizualize the difference in chromothripsis prediction between the 2 methods from bootstrap

```
hist(deltaV)
```

# Statistical significance of the difference in chromothripsis prediction between the 2 methods

```
sds = sd(deltaV)
pvalue = 2*(1-pnorm(abs(deltaObs)/sds))
pvalue
```

```
## [1] 0.000000000001048
```
