# Supplementary material for: Copy number signatures predict chromothripsis and clinical outcomes in newly diagnosed multiple myeloma
Source: Nat Commun. 2021 Aug 27;12:5172. doi: 10.1038/s41467-021-25469-8 (PMC8397708; doi:10.1038/s41467-021-25469-8)
Supplement: Supplementary file 8 — Reporting summary [file 41467_2021_25469_MOESM8_ESM.pdf]

nature research

Corresponding author(s): Francesco Maura

Last updated by author(s): 06/24/2021

Reporting Summary

Nature Research wishes to improve the reproducibility of the work that we publish. This form provides structure for consistency and transparency in reporting. For further information on Nature Research policies, see Authors & Referees and the Editorial Policy Checklist .

Statistics

For all statistical analyses, confirm that the following items are present in the figure legend, table legend, main text, or Methods section.

n/a	Confirmed	
☐	☒	The exact sample size (*n*) for each experimental group/condition, given as a discrete number and unit of measurement
☐	☒	A statement on whether measurements were taken from distinct samples or whether the same sample was measured repeatedly
☐	☒	The statistical test(s) used AND whether they are one- or two-sided<br>*Only common tests should be described solely by name; describe more complex techniques in the Methods section.*
☐	☒	A description of all covariates tested
☐	☒	A description of any assumptions or corrections, such as tests of normality and adjustment for multiple comparisons
☐	☒	A full description of the statistical parameters including central tendency (e.g. means) or other basic estimates (e.g. regression coefficient) AND variation (e.g. standard deviation) or associated estimates of uncertainty (e.g. confidence intervals)
☐	☒	For null hypothesis testing, the test statistic (e.g. *F*, *t*, *r*) with confidence intervals, effect sizes, degrees of freedom and *P* value noted<br>*Give P values as exact values whenever suitable.*
☒	☐	For Bayesian analysis, information on the choice of priors and Markov chain Monte Carlo settings
☒	☐	For hierarchical and complex designs, identification of the appropriate level for tests and full reporting of outcomes
☒	☐	Estimates of effect sizes (e.g. Cohen's *d*, Pearson's *r*), indicating how they were calculated

Our web collection on statistics for biologists contains articles on many of the points above.

Software and code

Policy information about availability of computer code

Data collection	Publicly available calls were used for all whole genome sequencing (WGS) data.
Data analysis	Somatic variant calling was performed using DELLY (v0.7.6)16 and Manta (v.1.5.0). Mutational signatures were analyzed by hdp (https://github.com/nicolaroberts/hdp) and mmsig (https://github.com/evenrus/mmsig). Copy number variation was analyzed using tCoNuT (https://github.com/tgen/tCoNuT) and controlFREEC in WGS, and FACETS (https://github.com/mskcc/facets) in WES.<br>The LOH_index was calculated using signature.tools.lib (https://github.com/Nik-Zainal-Group/signature.tools.lib), the GSS was calculated scarHRD (https://github.com/sztup/scarHRD).<br>Copy number signatures were extracted using hdp (https://github.com/nicolaroberts/hdp)

For manuscripts utilizing custom algorithms or software that are central to the research but not yet described in published literature, software must be made available to editors/reviewers. We strongly encourage code deposition in a community repository (e.g. GitHub). See the Nature Research guidelines for submitting code & software for further information.

Data

Policy information about availability of data

All manuscripts must include a data availability statement. This statement should provide the following information, where applicable:

- Accession codes, unique identifiers, or web links for publicly available datasets
- A list of figures that have associated raw data
- A description of any restrictions on data availability

Sequence files are available at the European Genome-phenome and dbGaP archive under the Accession codes:
• EGAD00001003309 and phs000348.v2.p1 WGS: 24 NDMM and 1 high risk smoldering multiple myeloma patient
• phs000748.v1.p1: WES and low coverage/long insert WGS sequencing data from 752 NDMM patients (CoMMpass trial; IA 13)
• EGAS00001004467: WGS data from 3 MM, 1 SMM, 1 PCL and 4 MGUS patients

- EGAD00001005028 WGS data from 3 therapy related AML patients.
- Pan-Cancer Analysis of Whole Genomes (PCAWG) study was accessed via the data portal: https://dcc.icgc.org/

Field-specific reporting

Please select the one below that is the best fit for your research. If you are not sure, read the appropriate sections before making your selection.

[x] Life sciences [ ] Behavioural & social sciences [ ] Ecological, evolutionary & environmental sciences

For a reference copy of the document with all sections, see nature.com/documents/nr-reporting-summary-flat.pdf

Life sciences study design

All studies must disclose on these points even when the disclosure is negative.

Sample size	In this study we used n=752 WGS samples from MM patients, n=269 WGS from several hematological cancers, and n=677 whole exome sequencing samples from MM patients.
Data exclusions	NA
Replication	All the sequencing data and bio-informatics codes are available in public depository.
Randomization	The study was not an experimental clinical treatment trial and hence no randomization was performed.
Blinding	NA

Reporting for specific materials, systems and methods

We require information from authors about some types of materials, experimental systems and methods used in many studies. Here, indicate whether each material, system or method listed is relevant to your study. If you are not sure if a list item applies to your research, read the appropriate section before selecting a response.

Materials & experimental systems

n/a	Involved in the study	
[x]	[ ]	Antibodies
[x]	[ ]	Eukaryotic cell lines
[x]	[ ]	Palaeontology
[x]	[ ]	Animals and other organisms
[ ]	[x]	Human research participants
[x]	[ ]	Clinical data

Methods

n/a	Involved in the study	
[x]	[ ]	ChIP-seq
[x]	[ ]	Flow cytometry
[x]	[ ]	MRI-based neuroimaging

Human research participants

Policy information about studies involving human research participants

Population characteristics	In this study, involving the use of human samples, samples and data were obtained and managed in accordance with the Declaration of Helsinki.
Recruitment	The recruitment was based on sample availability
Ethics oversight	MMRF CoMMpass study, Pan-Cancer ICGC Analysis of Whole Genomes study.

Note that full information on the approval of the study protocol must also be provided in the manuscript.
